# Comparative Transcriptomics and Metabolites Analysis of Two Closely Related *Euphorbia* Species Reveal Environmental Adaptation Mechanism and Active Ingredients Difference

**DOI:** 10.3389/fpls.2022.905275

**Published:** 2022-05-31

**Authors:** Han Zheng, Mu-Yao Yu, Yang Han, Badalahu Tai, Sheng-Fa Ni, Rui-Feng Ji, Chun-Juan Pu, Kang Chen, Fu-Quan Li, Hua Xiao, Ye Shen, Xiu-Teng Zhou, Lu-Qi Huang

**Affiliations:** ^1^State Key Laboratory of Dao-di Herbs, National Resource Center for Chinese Materia Medica, China Academy of Chinese Medical Sciences, Beijing, China; ^2^Mongolian Medicine College, Inner Mongolia Minzu University, Tongliao, China; ^3^Anhui University of Science and Technology, Huainan Xinhua Hospital, Huainan, China; ^4^Hulunbeier Mongolian Medical Hospital, Hulunbeier, China

**Keywords:** *Euphorbia*, comparative transcriptomics, metabolome, environmental adaptation, diterpenoid, EST-SSR

## Abstract

Roots of *Euphorbia fischeriana* and *Euphorbia ebracteolata* are recorded as the source plant of traditional Chinese medicine “Langdu,” containing active ingredients with anticancer and anti-AIDS activity. However, the two species have specific patterns in the graphic distribution. Compared with *E. ehracteolata, E. fischeriana* distributes in higher latitude and lower temperature areas and might have experienced cold stress adaptation. To reveal the molecular mechanism of environmental adaptation, RNA-seq was performed toward the roots, stems, and leaves of *E. fischeriana* and *E. ehracteolata*. A total of 6,830 pairs of putative orthologs between the two species were identified. Estimations of non-synonymous or synonymous substitution rate ratios for these orthologs indicated that 533 of the pairs may be under positive selection (Ka/Ks > 0.5). Functional enrichment analysis revealed that significant proportions of the orthologs were in the TCA cycle, fructose and mannose metabolism, starch and sucrose metabolism, fatty acid biosynthesis, and terpenoid biosynthesis providing insights into how the two closely related *Euphorbia* species adapted differentially to extreme environments. Consistent with the transcriptome, a higher content of soluble sugars and proline was obtained in *E. fischeriana*, reflecting the adaptation of plants to different environments. Additionally, 5 primary or secondary metabolites were screened as the biomarkers to distinguish the two species. Determination of 4 diterpenoids was established and performed, showing jolkinolide B as a representative component in *E. fischeriana*, whereas ingenol endemic to *E. ebracteolate*. To better study population genetics, EST-SSR markers were generated and tested in 9 species of *Euphorbia*. A total of 33 of the 68 pairs were screened out for producing clear fragments in at least four species, which will furthermore facilitate the studies on the genetic improvement and phylogenetics of this rapidly adapting taxon. In this study, transcriptome and metabolome analyses revealed the evolution of genes related to cold stress tolerance, biosynthesis of TCA cycle, soluble sugars, fatty acids, and amino acids, consistent with the molecular strategy that genotypes adapting to environment. The key active ingredients of the two species were quantitatively analyzed to reveal the difference in pharmacodynamic substance basis and molecular mechanism, providing insights into rational crude drug use.

## Introduction

*Euphorbia* is the largest genus in the Euphorbiaceae family, consisting of more than 2,000 species, with 77 species distributed in China (Li et al., 2008). Roots of *Euphorbia fischeriana* Steud (Li et al., 2021b) and *Euphorbia ebracteolata* Hayata (Yang et al., 2021) were used as the same kind of traditional Chinese medicine “Lvru” (now known as Langdu) for more than 2,000 years of treating swelling and ulcer of scabies (Zhao et al., 1996). As main active ingredients of Langdu, jolkinolide B (abietane type, multicyclic diterpenoid) and its derivatives have been proven to display potent anticancer activity (Wang et al., 2009, 2017a); ingenol's esters (ingenane type, bicyclic diterpenoid) have a great potential in treating human immunodeficiency virus (HIV) (Miana et al., 2015; Huang et al., 2019) and actinic keratosis (Parker et al., 2017), such as prostratin, Picato.

Although *E. fischeriana* and *E. ehracteolata* have been used interchangeably, the graphic distribution pattern of these two Langdu species is different (Li et al., 2008). Compared with *E. ehracteolata* growing in eastern and central China, *E. fischeriana* growing in northern and northeast China (higher latitude) has the habitat characteristics of lower annual average temperature (data from www.nmc.cn). In particular, *E. fischeriana* will be subjected to a strong cold stress in winter. Under this long-term environmental factor, the two species exhibit typical patterns of adaptive evolution and explosive speciation. From a genetic perspective, during speciation among closely related species, genes evolving rapidly are more differentiated than the rest, which is thought to be responsible for habitat differentiation and adaptation (Zhang et al., 2013; Mao et al., 2016; Zhao et al., 2016). However, few genomic resources are available for *E. fischeriana* and *E. ebracteolata*, leading to the unavailability of positive selection gene detection and the study of important loci. A few studies have been reported toward mechanism of ontogenesis (Prenner and Rudall, 2007), evolution of major structural characters (Horn et al., 2012), and diversity of species (Frajman et al., 2019) in *Euphorbia* (Euphorbiaceae), but neither of the two Langdu species was involved. Although the transcriptome of *E. fischeriana* has been sequenced (Barrero et al., 2011), it is not enough to explain the adaptive growth. With paucity of genetic data such as genome sequences and associated molecular markers, stress resistance or evolutionary analysis toward Langdu remains a challenge. In addition, studies have reported that the accumulation of compounds including soluble sugar, amino acids, and fatty acids contributes to adaptation toward abiotic stress (Wanner and Junttila, 1999; Duan et al., 2012; Wei et al., 2018). So far, chemical studies on Langdu have only been conducted on the active components of the drug, and few studies have been conducted on the compounds coping with stress resistance.

Transcriptome, characterized by its low cost and high efficiency, can not only provide additional genome resources and information about the process of speciation or adaptive evolution, such as time estimation of divergence, or detection of adaptive genes (Koenig et al., 2013; Zhao et al., 2018), but also provide expressed sequence tag—simple sequence repeat (EST-SSR) markers for species identification and germplasm evaluation (Varshney et al., 2005; Zhao et al., 2019; Li et al., 2021a). Furthermore, using metabolomics to study the metabolic changes under stress can reveal the response mechanism of plants to the changes in external environment or genes (Fiehn, 2002; De Vos et al., 2007). Therefore, we should not only reveal the molecular mechanism of adaptation to abiotic stress, but also study the effect of metabolite accumulation on adaptation to abiotic stress and the difference in active ingredients between the two plants. As the first step toward answering these questions, we obtained transcripts and metabolites for *E. fischeriana* and *E. ebracteolata*, furthermore carrying out a comprehensive analysis. 1) Transcripts of two Langdu species were identified, and their genetic differences were compared, providing additional genetic resources for Langdu breeding or evolutionary analysis; 2) evolutionary dynamics of two species were determined, obtaining an estimated time of differentiation, as well as the characteristics of adaptive evolution between the two species; 3) the differential metabolites of the two species were identified through metabolomics, and the chemical mechanism of adaptation toward stress was analyzed; 4) A UPLC-MS/MS method determining 4 diterpenoids simultaneously was established to detect diterpenoid contents in root and leaf of the two Langdu species; 5) and genus-specific EST-SSR markers based on the two species of Langdu were developed in preliminarily.

## Materials and Methods

### Plant Materials

We collected two Langdu species at fructescence. *E. fischeriana* is from hulun buir (48°34′57.18″N, 119°54′07.56″E, alt.746 m, Inner Mongolia); *E. ebracteolata* is from Jiyuan (35°12′46.56″N, 112°25′55.58″E, alt.708 m, Henan). Fresh leaves, stems, and roots from twelve individual plants for both species were stored in liquid nitrogen until total RNA extraction and metabolites analysis.

### Microscope Observation

Roots of *E. fischeriana* and *E. ebracteolata* were first embedded with paraffin and then cut into slices of 5 μm. Slices were dewaxed using xylene for 20 min two times, ethanol for 5 min two times, and 75% ethanol for 5 min and then washed with water. Dewaxed slices were soaked in saffron dye for 2 h and decolored with 50, 70, and 80% ethanol solution for 5 s. Fast green and ethanol were then used to dye and decolor, respectively. Slices were permeabilized using xylene and mounted with neutral gum. Microscopic features were captured using an Olympus BX51 microscope.

### RNA-Seq and Data Analysis

To provide a preliminary indication of genetic variation within species, the high-quality mRNA from leaves, stems, and roots from three individual plants for each species was isolated using Plant RNA Purification Reagent (Invitrogen, Life Technologies, USA) following the manufacturer's protocol. Both RNA-seq library preparation and paired-end sequencing were performed using an Illumina HiSeq 6000 platform (Zheng et al., 2021). All unigenes were first subjected to BLASTX (Altschul et al., 1997) against the National Center for Biotechnology Information (NCBI), including the non-redundant protein (Nr) database and non-redundant nucleotide sequence (Nt) databases with an *E*-value threshold of 10^−5^. The predicted gene name for each contig was assigned according to the best BLASTX hit. Protein structure and function were annotated with Swiss-Prot and Protein family (Pfam) database (Finn et al., 2010). Based on the annotations in NR, BLAST2GO v2.5 (Conesa et al., 2005) was used to obtain GO annotations for the aligned unigene sequences with an *E*-value threshold of 10^−6^, and the Web Gene Ontology Annotation Plot (WEGO) software (Ye et al., 2006) was used to establish GO functional classifications for all unigenes. The unigenes were aligned to the euKaryotic Ortholog Groups/Cluster of Orthologous Groups (KOG/COG) database to predict and classify possible functions, and the Kyoto Encyclopedia of Genes and Genomes (KEGG) pathways database (Kanehisa et al., 2008) was used to obtain pathway annotations (*E-*value threshold 10^−10^).

### Identification of Gene Orthologous Groups and Calculation of Ka/Ks

The Coding sequence (CDS) of each putative unigene were extracted according to the BLASTX results, and the orientation of the unaligned sequences was determined using ESTScan software. The CDSs extracted from the respected unigene were translated into amino acid sequences using the standard codon table. Self-to-self BLASTP was conducted for all amino acid sequences with a cutoff *E*-value of 10^−5^. Based on the predicted CDS regions of both *E. ehracteolata* and *E. fischeriana* transcriptomes, ORTHOMCL version 2.0.9 (Li et al., 2003) with default settings was used to reconstruct the clusters of orthologous groups (COGs). Pairs of putative orthologous genes were identified based on the reciprocal best matches with an *E*-value threshold of 10^−20^. Only pairs of sequences that mapped unambiguously to the same protein in Swiss-Prot database were selected as orthologous genes. The protein-coding sequences with unexpected stop codons in the BLAST hit region and/or shorter than 150 bp in length were removed.

Then, the Ks value of the obtained orthologous genes and the formula T = K/2r were used to estimate divergence time (T) between *E. fischeriana* and *E. ehracteolata* (Graur et al., 2000). “K” is a genetic divergence expressed in terms of mean number of synonymous substitutions between orthologs; “r” is the mean rate of synonymous substitution and is considered to be 1.5 e^−8^ substitutions/synonymous site/year for all dicots (Koch et al., 2000). Ka/Ks calculation was performed with PAML package (Yang, 2007) using default settings. Based on the Ka/Ks value and a threshold at 0.5, the orthologs were sub-categorized into two datasets: a test set with Ka/Ks above 0.5, and a reference dataset with Ka/Ks value < 0.5. The significance of the difference in GO term abundance between the two datasets was tested using the Fisher's exact test with the GOSSIP package (Bluthgen et al., 2005) implemented in BLAST2GO V.2.6 (Conesa et al., 2005).

### Metabolome Analysis

About 50 mg of fresh pulverized roots and leaves of *E. fischeriana* and *E. ehracteolata* was accurately weighed and extracted with 1.0 ml 80% methanol for LC-MS. Extraction was done at room temperature (RT) for 3 h in an orbital shaker set at 220 rpm. Resulting extracts were spun down at 3,000 *g* for 15 min to sediment tissue material. About 200 μl of supernatant was transferred to vial for LC-MS analysis using an established LC-MS-based approach (Su et al., 2016; Kong et al., 2017). The LC-MS detection data were extracted and preprocessed using SIEVE software (Thermo), and the data were normalized and later edited in Excel 2013. Finally, it is organized into a two-dimensional data matrix form, which contains information such as retention time (RT), compound molecular weight (CompMW), observations (sample name), amount of material extracted (ID), and peak intensity. A total of 1,220 features at (ESI+) ion mode and 1,506 features at (ESI-) ion mode after editing were performed multivariate analysis (MVA) using SIMCA-P software (version 13.0, Umetrics AB, Umea, Sweden). Principal component analysis (PCA) and partial least squares-discriminate analysis (PLS-DA) were employed to identify biochemical patterns. The metabolites that differed between the two classes were quantified using a combination of VIP statistics (threshold > 1) of the OPLS-DA model and *t*-tests (*p* < 0.05). Compounds were identified by a comparison of m/z or precise molecular mass at http://metlin.scripps.edu.

### Determination of Secondary Metabolites

Roots and leaves of *E. fischeriana* and *E. ehracteolata* were first freeze-dried then pulverized into powder. About 0.15 g of the fine powder was accurately weighed and extracted with 15 ml ethanol in an ultrasonic water bath at 30°C for 30 min. After cooling to room temperature, the extraction solution was filtered through a 0.22-μm millipore filter and injected directly into the UPLC system for analysis. Jolkinolide A (ChemFaces, CAS:37905-07-0, lot no: CFS201902), jolkinolide B (Nature Standard, CAS:37905-08-1, lot no:4069), jolkinolide E (ChemFaces, CAS:54494-34-7, lot no: CFS201901), and ingenol (Nature Standard, CAS:30220-46-3, lot no:3925) were used as the standard substances and were dissolved in ethanol to obtain stock solutions of 1 mg/ml^−1^. A number of 4 reference solutions were mixed and diluted with ethanol to obtain a series of mixture solutions. All the solutions were stored at −20°C before use.

Quantitation of active components was performed on an ultra-high-performance liquid chromatography coupled with tandem mass spectrometry (UPLC-MS/MS, LCMS 8045, Shimadzu, Japan). An ACQUITY BEH C_18_ column (2.1 mm × 100 mm, 1.7 μm) was used to separate diterpenoids. The mobile phase was composed of formic acid in water (0.05%, V/V) as solvent A and formic acid in ACN (0.05%, V/V) as solvent B, with a flow rate of 0.4 ml/min^−1^ at 40°C. The optimized gradient elution was as follows: 0–6 min, 30–93%B; 6–9 min, 93%B; 9–9.2 min, 93–30%B. About 1 μl of the solution was injected. The mass system was equipped with an electrospray ionization (ESI) source operating in both positive and negative ion modes, using multiple reaction monitoring (MRM) mode. Optimized parameters are listed in Supplementary Table S1. The nebulizer and drying gas were 99.95% nitrogen, and their flow rates were 3.0 and 10.0 L/min^−1^, respectively. The heating gas was 99.95% air with a flow rate of 10.0 L/min^−1^. The collision gas was 99.99% argon with a pressure of 270 kPa. Other parameters were as follows: interface voltage 4.0 kV, interface temperature 300°C, desolvation line temperature 250°C, and heat block temperature 400°C. The precision [relative standard deviation (RSD) < 5.77%] and accuracy (recovery was from 95.9 to 105.1%) of this method met the requirements for quantitative determination. Linearity was verified using coefficients of determination (R2), which were all > 0.999 within the adopted linear range (Supplementary Material).

### Development and Detection of EST-SSR Markers

The B MISA (http://pgrc.ipk-gatersleben.de/misa/) (Dieringer and Schlötterer, 2003) was used to identify and localize microsatellite motifs in the two Langdu species and SSRs were considered to contain motifs of two to six nucleotides and a minimum of five contiguous repeat units. The alignments of 6,153 pairs of orthologs were extracted as the input file for the MISA program. Using the detailed information on SSR loci obtained from the output of the MISA program, primers for each SSR-containing sequence with a repetitive at least 15 bp in length were designed with Program Primer Premier 5 (PREMIER Biosoft Int., Palo Alto, CA). To validate the SSRs identified *in silico* identified SSRs, 68 primer pairs shared between the two Langdu species were synthesized [Sangon Biotech (Shanghai) Co., Ltd., Shanghai, China] and validated by polymerase chain reaction (PCR) in 9 species, including *Euphorbia fischeriana* (Hailar, Inner Mongolia), *Euphorbia ebracteolata* (Jiyuan, Henan province), *Euphorbia lathyris* (Lijiang, Yunnan province)*, Euphorbia pekinensis* (Chuzhou, Anhui province), *Euphorbia sieboldiana* (Wang qing, Jilin province), *Euphorbia kansui* (Yongji, Shanxi Province), *Euphorbia humifusa* (Yongji, Shanxi Province), *Euphorbia helioscopia* (Jiyuan, Henan province), and *Euphorbia esula* (Ewenke, Inner Mongolia autonomous region). According to the Flora of China (Li et al., 2008), samples are all commonly used Chinese medicine. Fresh leaves were dried in silica gel at the time of collection. Voucher specimens were deposited in National Resource Center for Chinese Materia Medica (Beijing, China).

Genomic DNA was extracted from the dry leaves using Plant Genomic DNA Kit (TIANGEN). PCRs were performed in a 25 μl volume containing 50 ng of template genomic DNA. The PCRs were carried out under the following conditions: initial denaturation at 94°C for 2 min, 35 cycles at 94°C for 30 s, 55°C for 30 s, 72°C for 40 s, and a final extension at 72°C for 10 min. The separation of alleles was performed on a 3% polyacrylamide gel with a 500-bp DNA marker (TaKaRa) to calculate the length of the EST-SSR amplicons.

## Results

### Differences in Habitats and Phenotypes Between the Two Langdu Species

**Figure 1:**
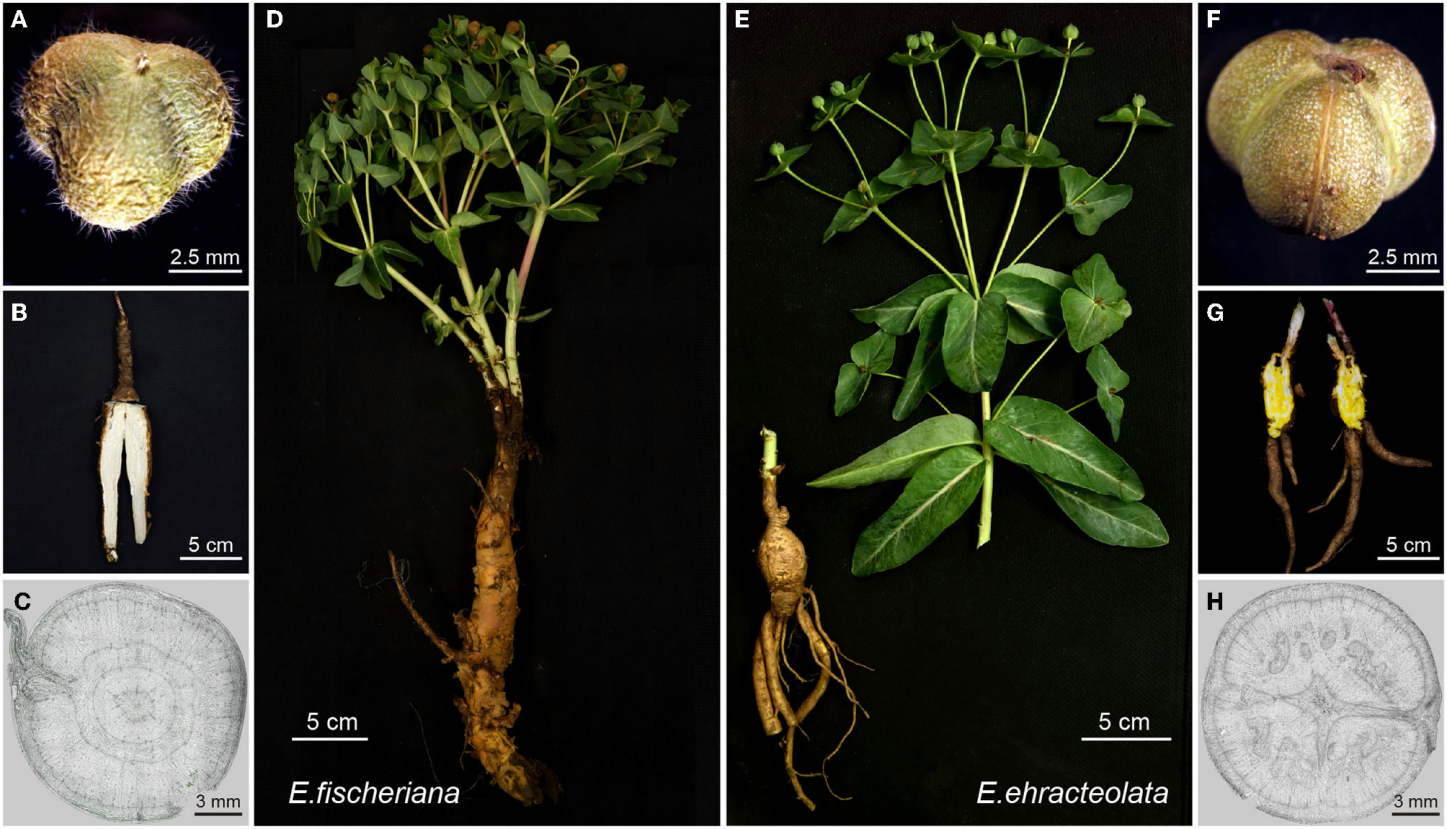
Two species of Langdu characteristic diagrams. **(A)** Fruit of *E. fischeriana*; **(B)** longitudinal section of *E. fischeriana* root; **(C)** cross-section micrograph of *E. fischeriana* root; **(D)** whole *E. fischeriana* plant; **(E)** whole *E. ehracteolata* plant; **(F)** fruit of *E. ehracteolata*; **(G)** longitudinal section of *E. ehracteolata* root; **(H)** cross-section micrograph of *E. ehracteolata* root.

To study these two closely related species, we collected wild *E. fischeriana* in Hailar, Inner Mongolia and wild *E. ehracteolata* in Jiyuan, Henan province, which are the main producing areas of two species, respectively. The average annual temperature in Hailar is −2.0°C, and that in Jiyuan is 14.6°C (data from www.nmc.cn). Therefore, in terms of habitat distribution, *E. fischeriana* is more tolerant of coldness than *E. ehracteolata*. This adaptive distribution might also result in phenotypic differences between the two Langdu species. Classification of the two species majorly depends on whether the ovary and capsule have trichome (Figures 1A,F) (Li et al., 2008), but the identification and comparison of the experimental materials showed that their roots (medicinal part) are also significantly different. The root of *E. fischeriana* is elongated and enlarged with multiple layers of semi-exfoliated epidermis, whereas *E. ehracteolata* is fusiform and the outer epidermis is tight (Figures 1D,E). In root of *E. fischeriana*, latex is white (Figure 1B) with concentric ring vascular bundle (Figure 1C), whereas yellow latex (Figure 1G) and abnormal vascular bundle (Figure 1H) are obtained in *E. ebracteolata*. The medicinal materials from the two species are similar in appearance, mostly in transverse slices or oblique slices, collectively referred to as “white Langdu,” which is easy to be confused. Therefore, we further developed biomarkers to assist the identification of medicinal materials.

### *De novo* Assembly and Functional Annotation

To reveal the molecular mechanism of adaptive distribution, cDNA libraries of the root, stem, and leaf of *E. fischeriana and E. ehracteolata* were sequenced, with 169594954 and 145943522 raw reads obtained (Table 1). The average Q20 percentage (percentage of sequences with sequencing error rate lower than 1%) was 97.17 and 97.03%. The average GC percentage was 42.58 and 42.97%. Using the trinity *de novo* assembly program, we assembled the short read sequences from the three tissues into 149027 transcripts, with a mean length of 813 bp and an N50 length (the contig size such that 50% of the entire assembly is contained in contigs equal to or longer than this value) of 1,559 bp for the *E. fischeriana*. For *E. ehracteolata*, 143,857 contigs longer than 200 bp, with a mean length of 885 bp and an N50 length of 1,635 bp, were generated. In total, all contigs were connected into 112,487 unigenes with a mean length of 631 bp and an N50 of 1,051 bp for *E. fischeriana* and 101,211 unigenes with a mean length of 686 and an N50 value of 1,115 for *E. ehracteolata*.

**Table 1:** Summary of assembly results for *E. fischeriana* and *E. ehracteolata* using trinity.

**Sequence**	***E. fischeriana***			***E. ehracteolata***		
	**Root**	**Stem**	**Leaf**	**Root**	**Stem**	**Leaf**
Raw reads	50845208	46010830	49087484	61659938	56456010	51479006
Clean reads	49966652	45210234	48124262	60048398	54973026	50043212
Q20 (%)	97.09	97.07	96.92	97.17	97.2	97.13
GC (%)	43.54	42.53	42.83	42.62	42.62	42.51
Total number of contigs/unigenes	149,207/112,487			143,857/101,211		
Length range of contigs/unigenes	200–15,623			200–16,340		
N50 value of contigs /unigenes	1,559/1,051			1,635/1,155		
Mean length of contigs/unigenes	813/631			885/686		
Median length of contigs/unigenes	398/325			459/366		

**Figure 2:**
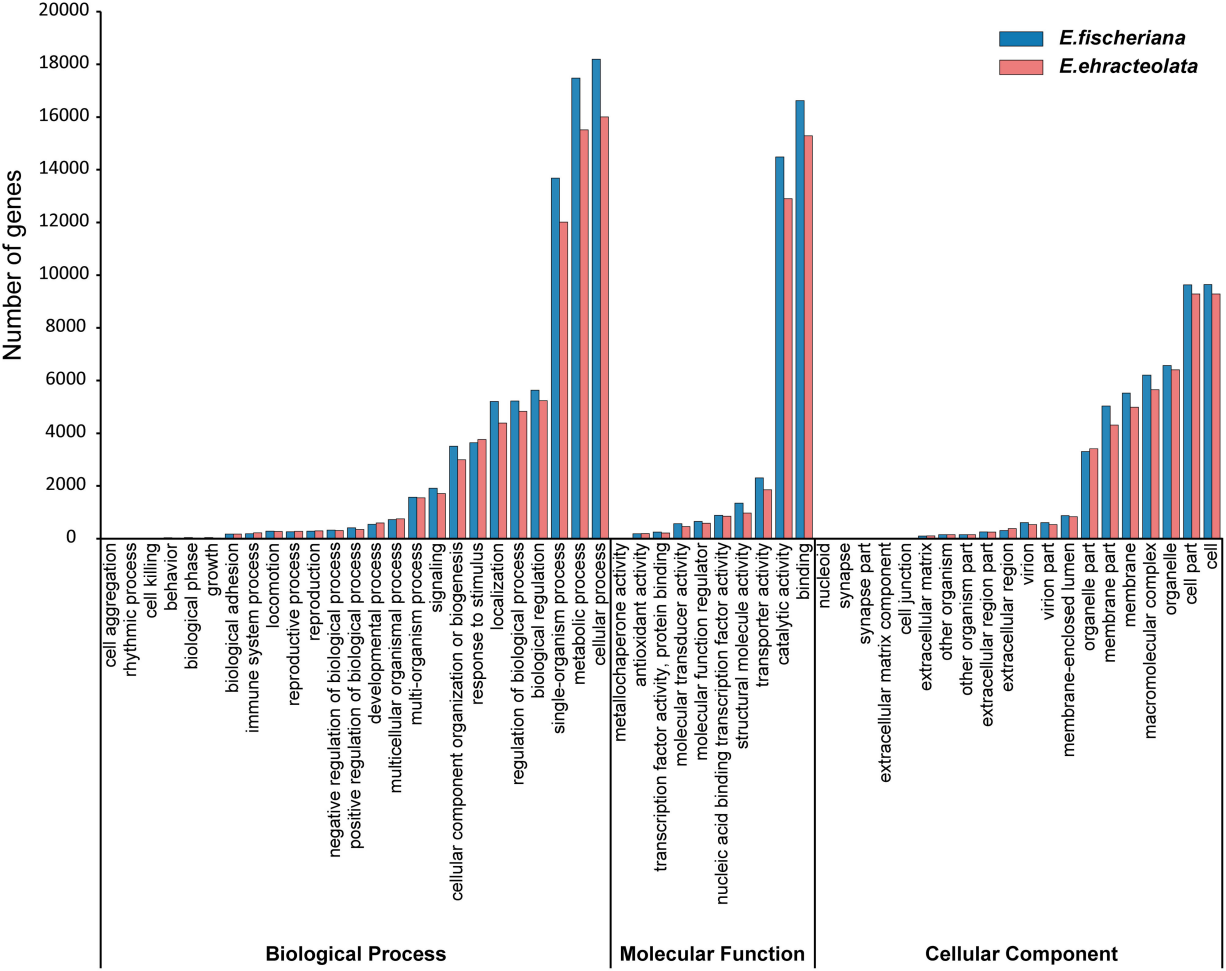
Functional annotation of assembled sequences based on gene ontology (GO) categorization.

To obtain comprehensive gene function information, we carried out gene function annotation of seven databases, including Nr, Nt, Pfam, KOG/COG, Swiss-Prot, KEGG, and GO (Supplementary Table S2). The all unigenes were assigned putative gene descriptions based on the BLAST (*E*-value ≤ 1 × 10e^−5^) search against the NCBI non-redundant (Nr) protein database. A total of 36,204 (32.18%) unigenes for *E. fischeriana* and 38,018 (37.56%) unigenes for *E. ehracteolata* were shown significant similarity with proteins in the Nr database. There were 48,033 (42.70%) unigenes for *E. fischeriana* and 44,919 (44.38%) for *E. ehracteolata* with at least one significant match to the above databases. For both species, a BLASTX top-hit species distribution of gene annotations showed highest homology to *Jatropha curcas* [*E. fischeriana* (38.49%) vs. *E. ehracteolata* (39.02%)]. Based on Nr annotations, we used the GO classification system to functionally categorize unigenes. A total of 28,472 (28.13%) unigenes for *E. ehracteolata* and 32,598 (28.97%) unigenes for *E. fischeriana* were assigned to at least one GO term annotation. The unigenes were assigned to three main GO categories (Figure 2): biological process (*E. ehracteolata*: 71,495, 47.30%; *E. fischeriana*: 79,428, 47.91%), molecular function (33,335, 22.06 vs. 37,307, 22.51%), and cellular component (46,314, 30.64 vs. 49,036, 29.58%). These categories were similarly distributed in both species.

For the biological process category, the two mostly highly represented terms among the 25 level-2 categories were cellular process, metabolic process, and single-organism process; for the molecular function category, among the 10 level-2 categories, binding and catalytic activity were overrepresented; for the 20 level-2 categories in the cellular component category, cell, cell part, and organelle were the most abundant terms (Figure 2). These categories were similarly distributed in both species. Specifically, compared with *E. ehracteolata*, more genes in *E. fischeriana* were involved in biological process and molecular function, including metabolic process (15,517, 10.27 vs. 17,479, 10.54%), localization (4,390, 2.91 vs. 5,209, 3.14%), cellular component organization or biogenesis (2,994, 1.98 vs. 3,508, 2.12%), positive regulation of biological process (351, 0.23 vs. 416, 0.25%), molecular transducer activity (451, 0.30 vs. 571, 0.34%), structural molecule activity (975, 0.65 vs. 1,347, 0.81%), transporter activity (1,853, 1.23 vs. 2,305, 1.39%), and so on. However, genes involved in biological phase (34, 0.02 vs. 42, 0.03%), growth (36, 0.02 vs. 46, 0.03%), immune system process (183, 0.11 vs. 226, 0.15%), and developmental process (544, 0.33 vs. 598, 0.40%) showed a conversed trend, comparing *E. fischeriana* with *E. ehracteolata*.

### Orthologous Genes and Substitution Rates Between Two Langdu Species

**Figure 3:**
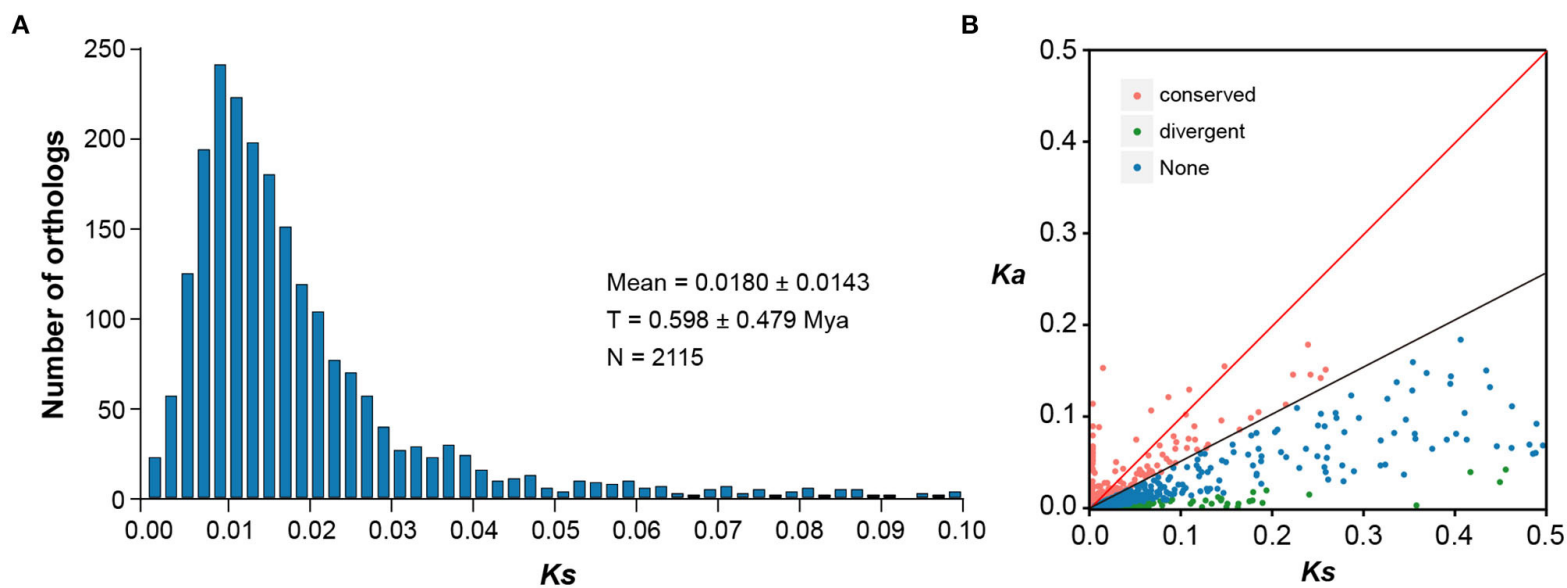
Distribution of Ka and Ks for 6,830 pairs of the putative orthologous genes between *E. fischeriana* and *E. ehracteolata*. **(A)** The Ks distribution of orthologs between *E. fischeriana* and *E. ehracteolata* and their divergence is shown by the peak of Ks at 0.018 ± 0.014. **(B)** The orthologs with *Ka*/*Ks*>1 fall above the red line whereas those with Ka/Ks = 0.5–1.0 fall between the black and red lines.

Based on the predicted CDS regions of both *E. fischeriana* and *E. ehracteolata* transcriptomes, we identified initial putative orthologous pairs. After removing the pairs with unexpected stop codons in the BLAST hit region and/or shorter than 150 bp in length, 6,830 ortholog pairs were retained. Out of the 6,830 ortholog pairs, 3,193 pairs had both non-synonymous (Ka) and synonymous (Ks) substitutions and thus were allowed for the calculation of Ka/Ks ratios. A peak of Ks value distribution between *E. fischeriana* and *E. ehracteolata* was observed at 0.0180 ± 0.0143 (Figure 3A), and the low level of Ks value indicates close relationship. It was estimated by the formula T = K/2r that the age of the speciation event between *E. fischeriana* and *E. ehracteolata* is ~0.598 Mya, which falls in the Middle Pleistocene. Among the 3,193 ortholog pairs, 533 pairs showed high Ka/Ks values (Figure 3B), in which 191 pairs have a Ka/Ks > 1 (*p* < 0.05), indicating positive selection, whereas 342 pairs have a Ka/Ks between 0.5 and 1, indicating weak positive selection. By contrast, most of the orthologous pairs (2,658) showed a Ka/Ks ratio < 0.5, indicating that these gene pairs are likely under purifying selection.

### Gene Functions Under Positive Selection and Implications for Adaptive Evolution Between Two Langdu Species

**Figure 4:**
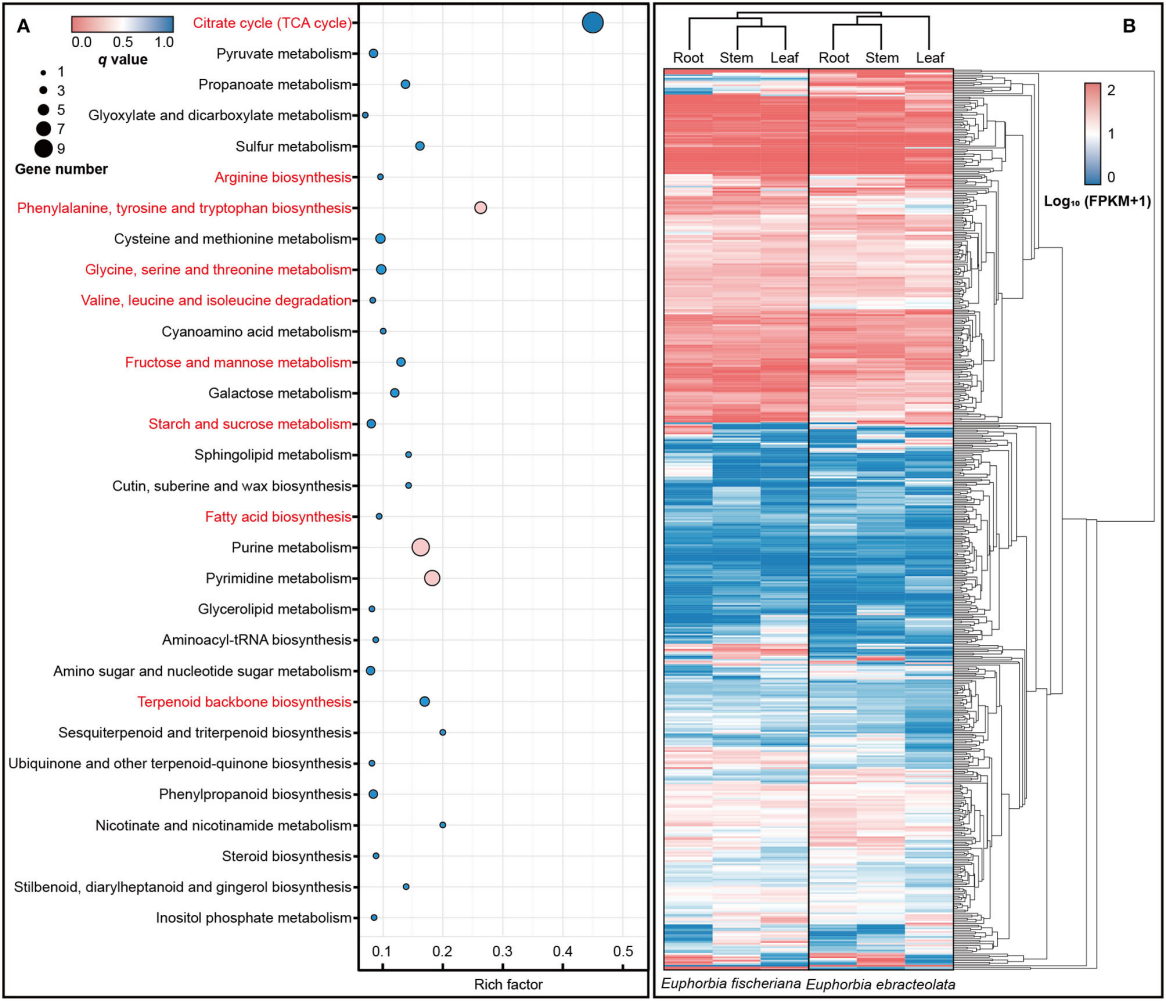
KEGG enrichment and expression differences of orthologous genes. **(A)** KEGG enrichment of orthologous genes (Ka/Ks > 0.5); **(B)** Heat map of orthologous genes (Ka/Ks > 0.5) expression in roots, stems, and leaves.

In enrichment analysis, we used the orthologs into one dataset with Ka/Ks > 0.5 (Swanson et al., 2004). In KEGG database, the positive selection orthologous genes were enriched to 57 pathways, including (1) citrate cycle (TCA cycle); (2) amino acid biosynthesis and metabolism including arginine biosynthesis, phenylalanine, tyrosine and tryptophan biosynthesis, cysteine and methionine metabolism, glycine, serine and threonine metabolism, as well as valine, leucine, and isoleucine degradation; (3) fructose and mannose metabolism; (4) starch and sucrose metabolism; (5) fatty acid biosynthesis; and (6) terpenoid backbone biosynthesis (Figure 4A). The expression levels of orthologous genes in pathway are also different in two Langdu species (Figure 4B).

In an analysis of GO terms with at least five hits, 31 GO terms annotated to 76 pairs of orthologs were found to be over-represented (*p*-value < 0.05) in the test dataset. For the selected genes, we used BLASTX search to find their orthologous genes, and genes with function in stress tolerance, development, TCA-related, and so on were filtered (Supplementary Table S3). In particular, half of terms involved genes were related to stress tolerance, including cold stress, heat stress, and drought stress. Terms containing cold stress tolerance gene accounted for 30%, including *AtGCN1* (Wang et al., 2017b), *AtYLS9* (Griebel et al., 2022), *BrRZFP* (Jung et al., 2013)*, AtPTP1* (Liu et al., 2016), and *AtPFD* (Perea-Resa et al., 2017). In terms of growth and development, we enriched genes related to trichome and root hair development, such as *AtBLT* (Kasili et al., 2011; Mazie and Baum, 2016), *AtVTI13* (Larson et al., 2014), *AtNRP2* (Zhu et al., 2017), and *AtWER1* (Wang et al., 2019). TCA-related genes were also enriched, including *AtSAM1* (Sekula et al., 2020), *AtLIP1* (Wang et al., 2022), *AtPES1* (Lippold et al., 2012), *AtCCDC* (Lohmeier-Vogel et al., 2008), and *AtBASS2* (Furumoto et al., 2011; Mueller et al., 2014; Lee et al., 2017), involving amino acid biosynthesis, catabolism of triacylglycerols, fatty acid, starch metabolism, pyruvate transport, etc.

### Identification of Metabolites Differentially Accumulated in Two Langdu Species

**Figure 5:**
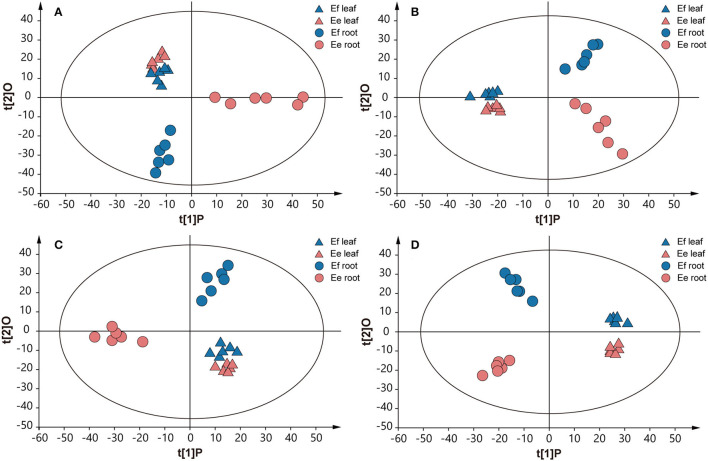
Score plots of PCA and OPLS-DA. **(A)** PCA in positive mode. **(B)** PCA in negative mode. **(C)** OPLS-DA analysis in positive mode. **(D)** OPLS-DA analysis in negative mode. Multivariate analysis of metabolic profiles of root and leaf samples from *E. fischeriana* and *E. ehracteolata* with 6 biological replicates.

Due to the above genetic differences, we further studied the metabolic differences between the two species, to reveal the effects of genotypes on chemical phenotypes. The chemical components in roots and leaves were detected through UPLC–MS analysis, obtaining final data containing 1,220 features in ESI+ mode and 1,506 features in ESI- mode. An unsupervised principal component analysis (PCA) model was used, since it can represent the intrinsic characteristics of the data. The result revealed a clear separation of metabolite samples between *E. fischeriana* and *E. ehracteolata* (R^2^X = 0.724, Q^2^ = 0.561 in ESI+ mode; R^2^X = 0.598, Q^2^ = 0.387 in ESI– mode) (Figures 5A,B). The PCA score plot revealed that the leaves from *E. fischeriana* (blue triangle) and *E. ehracteolata* (red triangle) are not perfectly separated by the 12 samples. Similar to the appearance of the two Langdu leaves, there is less difference in the composition and content of their leaves. But the roots of *E. fischeriana* (blue circle) and *E. ehracteolata* (red circle) were clearly separated. The results indicated that there were significant differences in the compounds of Langdu roots. The PCA model was also applied to obtain a preliminary overview of general similarities and differences between collections. To identify discriminating metabolites and differentiate the four groups, we used the corresponding OPLS-DA analysis (Figures 5C,D). The two Langdu species can be separated clearly by only one predictive component deriving from a more sophisticated orthogonal partial least squares discriminate analysis (OPLS–DA) model, since noisy information irrelevant to species was removed prior to model building (Bylesjo et al., 2006). OPLS–DA analysis showed potential markers in different Langdu plants. A total of 98 discriminating metabolites (VIP > 1.0, *p* < 0.05), including 46 in positive mode (R^2^X = 0.758, R^2^Y = 0.965, Q^2^ = 0.917) and 52 in negative mode (R^2^X = 0.593, R^2^Y = 0.981, Q^2^ = 0.926), were identified in Langdu roots. The permutation result validated the stability and reliability of this OPLS–DA model. Subsequently, relying on the three criteria—variable importance in the projection (VIP) value of OPLS–DA model ≥ 1.5, *p*-value of *t*-test ≤ 0.01–5 metabolites could be presumably considered as candidate biomarkers. In addition, jolkinolide B and ingenol showed specific accumulation in the two species and could also serve as the biomarkers (Supplementary Table S4).

**Figure 6:**
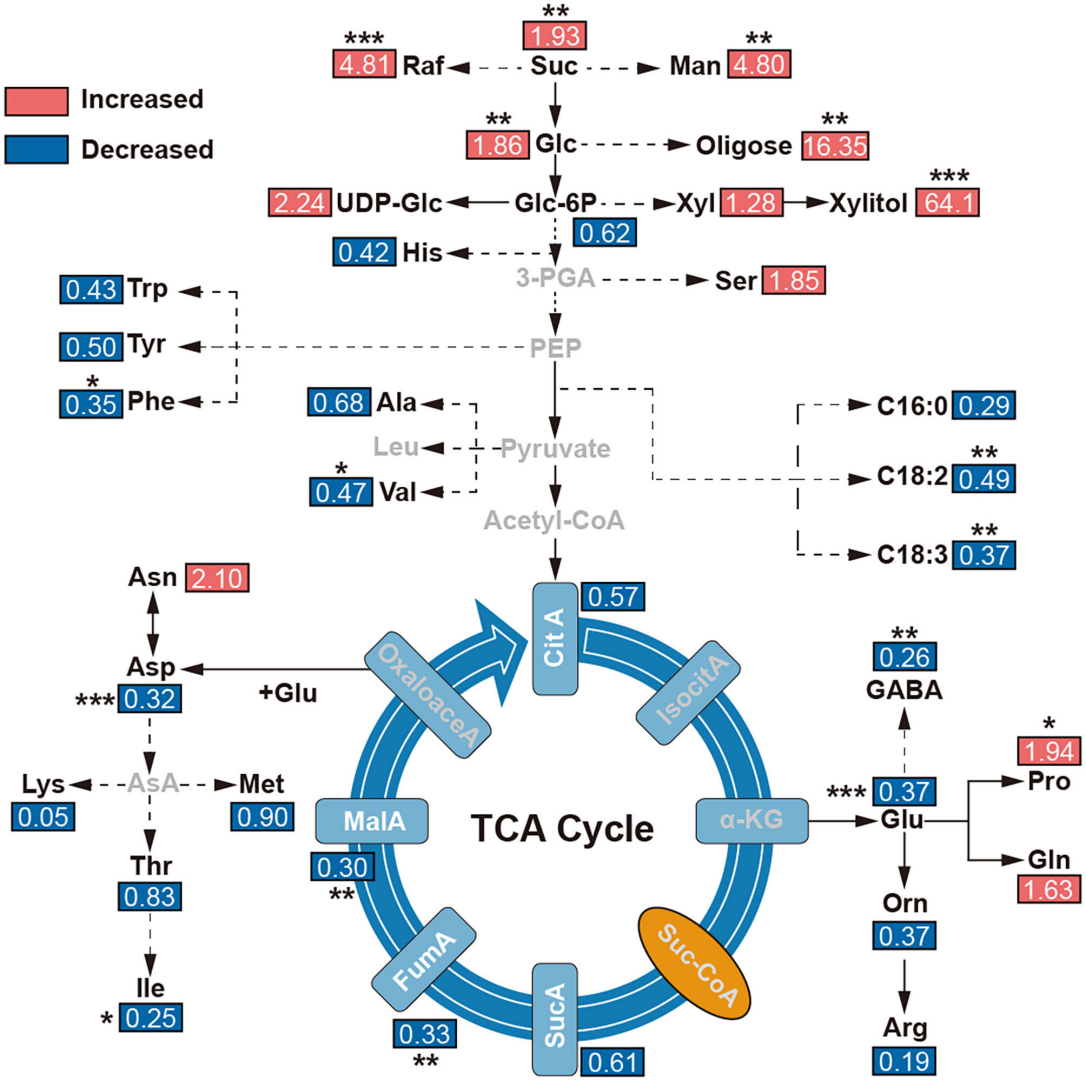
Levels of metabolites in main metabolic maps. The comparisons of metabolite contents were calculated by dividing the metabolite level in *E. fischeriana* with that in *E. ehracteolata*. 3-PGA, glycerate 3-phosphate; α-KG, alpha-ketoglutarate; Arg, arginine; ASA, aspartic acid- β-hemialdehyde, Asn, asparagine; Asp, aspartic acid; Ala, beta-alanine; CitA, Citric acid; PalA, palmitic acid; OctA, octadecanedioic acid; LinA, linoleic acid; α-Lin, α-linolenic acid; FumA, fumarate; GABA, γ-aminobutyric acid; Glc, glucose; Glc3P, glucose-3-phosphate; Gln, glutamine; Glu, glutamate; Gly, glycine; Ile, isoleucine; IsocitA, isocitric acid; Leu, leucine; Lys, lysine; MalA, malate; Man, mannitol; Met, methionine; Orn, ornithine; Pro, proline; Raf, raffinose; Ser, L-serine; Stigm, stigmasterol; Suc, sucrose; SucA, succinic acid; Suc-CoA, succinate coenzyme A; Thr, threonine; Trp, tryptophan; Val, valine; Xly, xylulose. The value represents the ratio of *E. fischeriana* to *E. ehracteolata*. Asterisks denote Student's *t*-test significance: *p < 0.05; **p < 0.01; ***p < 0.001.

To reveal the effect of environments on the metabolites, the tentatively identified compounds were assigned in the common metabolic pathways according to the literature works (Schauer et al., 2006; Duan et al., 2012). Combined with previous KEGG and GO enrichment results of adaptive evolution genes, we found that the contents of malic acid, fumaric acid, GABA, amino acids, and other compounds in TCA in *E. fischeriana* were significantly lower than in *E. ehracteolata*. But some soluble sugars, such as glucose, sucrose, raffinose, xylulose, and mannitol, were increased in *E. fischeriana* in comparison with the *E. ehracteolata*. Specifically, proline in *E. fischeriana* was significantly higher than that in *E. Ehracteolata*, but unsaturated fatty acids including linoleic acid and α-linolenic acid were lower (Figure 6).

### Differences in Expression Levels of Orthologous Genes and Active Ingredients

The differences in active ingredients between two Langdu species may result from the differences in genotype and gene expression. *DXR* (OG06739) and *MK* (OG07880) were found in the positive selection library (Ka/Ks > 0.5) mentioned in the previous study, indicating that these two genes had adaptive mutations. We also analyzed the expression levels of MVA and MEP pathways and terpene synthase homologous genes globally and found that the gene expressive abundance of *E. fischeriana* was higher than that of *E. ehracteolata*, especially *DXS, DXR, CMK, HDS, HDR, HMGS, HMGR, MK*, and diterpene synthase genes *CPS* and *KSL* in the roots. However, the expression level of *CBS* in *E. ehracteolata* root was significantly higher than that of *E. fischeriana*, and even *CBS* (OG17313) was not expressing in *E. fischeriana* root.

**Figure 7:**
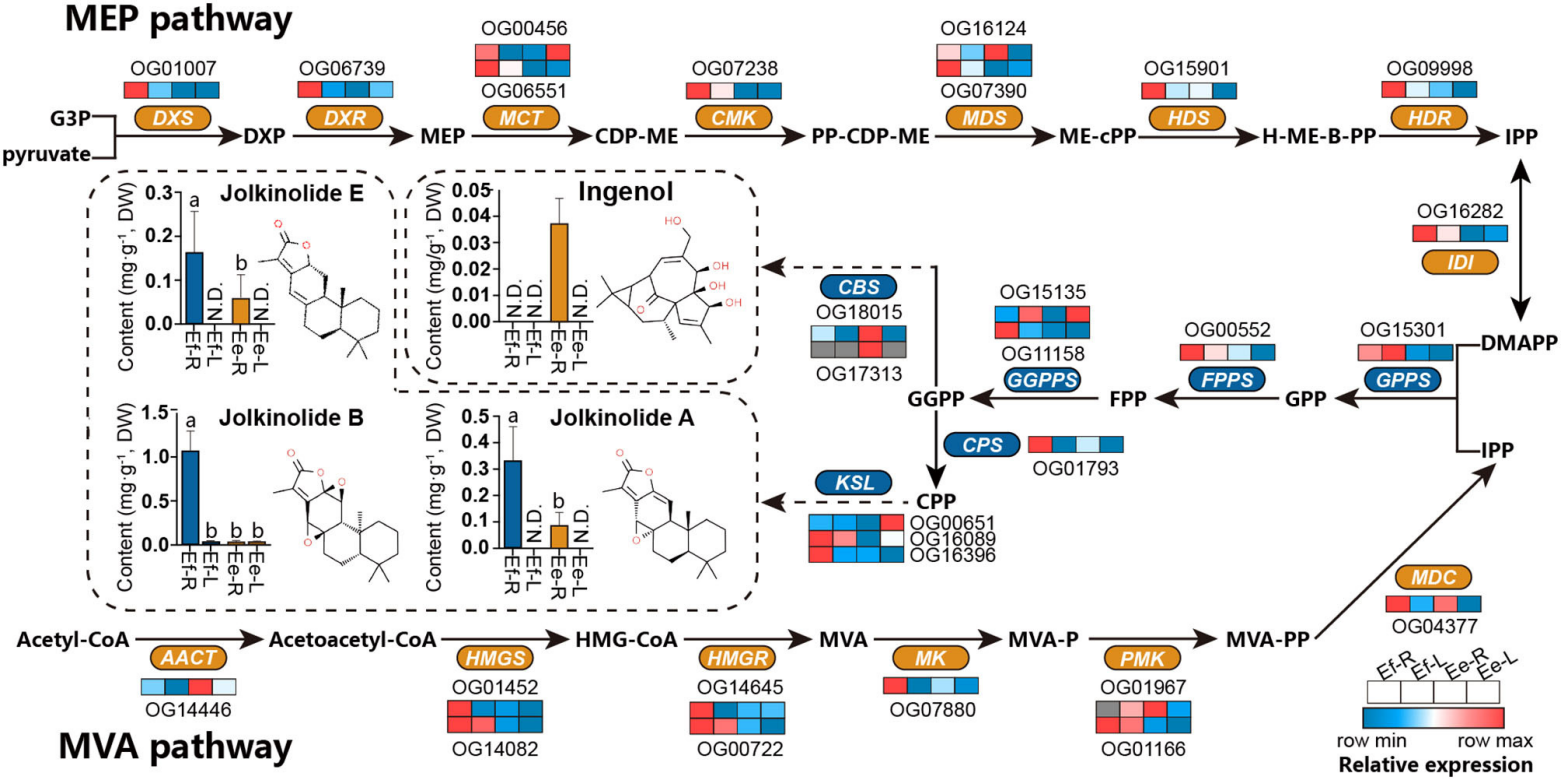
Differences in active ingredients and the expression levels of orthologous genes in *E. fischeriana* and *E. ehracteolata*. MVA pathway, the mevalonate pathway; Acetyl-CoA, acetyl coenzyme A; acetoacetyl-CoA, acetoacetyl coenzyme A; HMG-CoA, 3-hydroxy-3-methylglutaryl CoA; MVA, mevalonate; MVA-P, mevalonate-5-phosphate; MVA-PP, mevalonate-5-diphosphomevalonate; IPP, isopentenyl pyrophosphate; MEP pathway, the 2-C-methyl- d -erythritol 4-phosphate pathway; G3P, glyceraldehyde 3-phosphate; DXP, 1-deoxy-d-xylulose 5-phosphate; MEP, 2-C-methyl-d-erythritol 4-phosphate; CDP-ME, 4-(cytidine 5'-diphospho)-2-C-methyl- d -erythritol; PP-CDP-ME, 2-phospho-4-(cytidine 5'-diphospho)-2-C-methyl-d-erythritol; ME-cPP, 2-C-methyl-d-erythritol 2,4-cyclodiphosphate; H-ME-B-PP, 1-hydroxy-2-methyl-2-butenyl 4-diphosphate; DMAPP, dimethylallyl pyrophosphate; GPP, geranyl diphosphate; FPP, farnesyl diphosphate; GGPP, geranylgeranyl diphosphate; CPP, copalyl diphosphate; AACT, acetoacetyl-CoA transferase; HMGS, hydroxymethyl-glutaryl-CoA synthase; HMGR, 3-hydroxy-3-methylglutaryl CoA reductase; MK, mevalonate kinase; PMK, phosphomevalonate kinase; MDC, mevalonate-5-pyrophosphate decarboxylase; DXS, 1-deoxy-d-xylulose-5-phosphate synthase; DXR, 1-deoxy-d-xylulose-5-phosphate reductoisomerase; MCT, 2-C-methyl-D-erythritol 4-phosphate cytidylyltransferase; CMK, 4-(cytidine 5'-diphospho)-2-C-methyl-d-erythritol kinase; MDS, 2-C-methyl-d-erythritol 2,4-cyclodiphosphate synthase; HDS, 4-hydroxy-3-methylbut-2-enyl diphosphate synthase; HDR: 4-hydroxy-3-methyl-but-2-enyl diphosphate reductase; IDI, Isopentenyl-diphosphate delta-isomerase; GPPS, GPP synthase; FPPS, FPP synthase; GGPS, GGPP synthase; CPS, copalyl diphosphate synthase; KSL, kaurene synthase-like; CBS, casbane synthase. N.D., below detection limit. Different letters denote statistically significant differences (ANOVA/LSD, *P* < 0.05).

To analyze the differences in active ingredients, a UPLC-MS/MS method simultaneously determining 4 diterpenoids was established, and the root and leaf tissues of the two Langdu species were detected (Figure 7). The content of jolkinolide A was about 0.31 mg/g^−1^ in the roots of *E. fischeriana* and 0.10 mg/g^−1^ in the roots of *E. ehracteolata*. Jolkinolide B was significantly accumulated in the roots of *E. fischeriana* (1.13 mg/g^−1^) and slightly accumulated in the leaves (0.03 mg/g^−1^), whereas the content of jolkinolide B in the roots of *E. ehracteolata* was 0.03 mg/g^−1^. Jolkinolide E reached 0.16 mg/g^−1^ in the root of *E. fischeriana* and 0.06 mg/g^−1^ in *E. ehracteolata*. Conversely, bicyclic diterpenoid ingenol was only detected in the roots of *E. ehracteolata*, about 0.04 mg/g^−1^. Obviously, there were the differences in active ingredients biosynthesis genes and their expression levels between the two species during the evolution of environmental adaptation, which resulted in the preference of accumulation.

### Development of EST-SSR Markers in *Euphorbia* Species

Based on the *E. fischeriana* and *E. ehracteolata* transcriptomes, 8,458 and 10,612 EST-SSRs were identified, respectively. The most common repeat types were dinucleotides (44.0 and 44.5%), followed by trinucleotides (42.8 and 43.3%), and the most common repeat motifs were AG/CT, AT/AT, and AC/GT, followed by AAG/CTT and AAT/ATT (Supplementary Table S5). To maximize the universal applicability of markers developed in this study and hence reduce their cost, we searched for EST-SSRs in the 6,153 pairs of putative orthologous unigenes and found 967 EST-SSRs distributed among 780 pairs of orthologous unigenes. Taking only those with a repeat unit length of at least 15 bp, 68 pairs of EST-SSRs contained in 532 pairs of orthologs were selected for primer design, and 68 pairs of sequences with conserved, sufficiently long flanking sites were used to design primers successfully. To evaluate the reliability and cross-species transferability of these primers, we tested all the 68 pairs of EST-SSR markers for 9 species of *Euphorbia*. The average commonality ratio of primers in species was 35.69%, 49 pairs produced clear fragments with the expected sizes in both two Langdu and two pairs (OG00193 and OG07454) which produced clear fragments with the expected sizes in all nine species, respectively. A total of nine pairs produced fragments in eight species (Supplementary Table S6). Among them, OG07421, OG10880, and OG13687 could be amplified with obvious polymorphism bands in several *Euphorbia* species.

## Discussion

### The Environmental Adaptation of Two Langdu Is Influenced by Genotype and Metabolites

**Figure 8:**
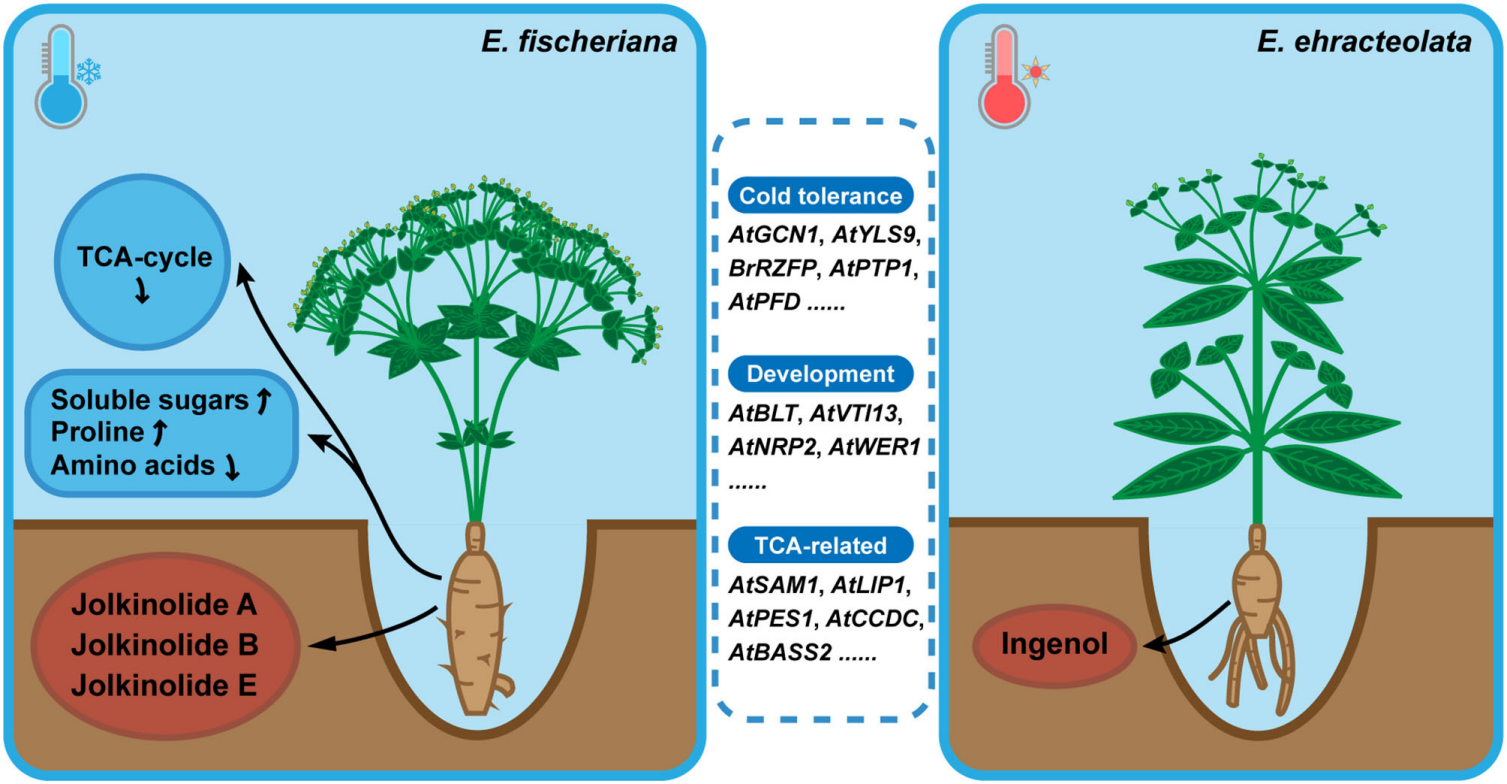
Model for environmental adaptation mechanism of Langdu species.Compared with *E. ehracteolata* growing in eastern and central China, *E. fischeriana* growing in northern and northeast China (higher latitude) has the habitat characteristics of lower annual average temperature, presenting more tolerance of coldness than *E. ehracteolata*. In *E. fischeriana, AtGCN1, AtYLS9, BrRZFP, AtPTP1, AtPFD* related to cold stress tolerance, *AtBLT, AtVTI13, AtNRP2, AtWER1* participated in trichome, root hair and epidermal development, *AtSAM1, AtLIP1, AtPES1, AtCCDC, AtBASS2* related to TCA-cycle were enriched. These differences in positive selection genes may ultimately affect phenotypes and metabolites accumulation. In addition, content of soluble sugars and proline was significantly higher in *E. fischeriana*, whereas content of amino acids was relatively lower, compared with that in *E. ehracteolata*. Differential accumulation of active ingredients also existed in two Langdu species, during the evolution of environmental adaptation. *E. fischeriana* preferred to accumulate polycyclic diterpenes such as jolkinolide A, jolkinolide B, and jolkinolide E, whereas *E. ebracteolata* preferred to accumulate dicyclic diterpenes such as ingenol, reflecting different accumulation strategies of the same metabolites in related species, which is the result of long-term adaptation to the environment and also the material basis for the formation of pharmacodynamics.

*E. fischeriana* mostly grows in northern and northeast China, with high latitude, less rainfall, low annual average temperature. However, *E. ehracteolata* mostly grows in eastern and central China (Li et al., 2008). Temperature, as one of the main environmental variables, makes that the two species exhibit a typical pattern of adaptive evolution and explosive speciation, which may be influenced by genotype and metabolites. The positive selection orthologous genes were enriched to TCA cycle, stress tolerance, development, amino acid biosynthesis and metabolism, fructose and mannose metabolism, starch and sucrose metabolism, and fatty acid biosynthesis pathways (Figure 8). Among them, OG06839 is homologous to *AtGCN1*, which mediated phosphorylation of eIF2α and is essential for cold tolerance (Wang et al., 2017b); OG13992 is homologous to *AtYLS9*, which tolerates cold stress by activating an immune response (Griebel et al., 2022); OG06917 is homologous to *BrRZFP*, which regulates germination, fresh weight, and length of shoots and roots to tolerate stress (Jung et al., 2013); OG01773 is homologous to *AtPTP1* and encodes a protein with tyrosine phosphatase activity that is downregulated in response to cold stress (Liu et al., 2016); OG07015 is homologous to *AtPFD* and controls the levels of HY5, a key role in cold acclimation by activating anthocyanin biosynthesis, in response to low temperature (Perea-Resa et al., 2017). The positive selection of the plant physiology-related genes may be one of the reasons why the two species can survive in different cold environments.

Trichomes play important protective roles as against abiotic stressors such as cold, drought, heat, excess of light, and UV radiation (Wagner et al., 2004; Hauser, 2014). For example, OG07214, which is homologous with *AtBLT*, may be a key gene in trichome development (Kasili et al., 2011; Mazie and Baum, 2016). The non-synonymous substitution of OG07214 may cause formation of trichome in *E. fischeriana* fruits (Figures 1A,F). In addition, *AtVTI13* (OG07291) is essential for the maintenance of cell wall organization and root hair growth (Larson et al., 2014); *AtNRP2* moderates chromatin structure for proper root hair development (Zhu et al., 2017). *AtWER1* plays an important role in generating the proper balance of downstream transcriptional factors in the gene regulatory network that establishes root epidermal cell fate (Wang et al., 2019). These gene differences may explain the larger root crown and more root epidermis of *E. fischeriana* (Figures 1D,E).

The accumulation of pyruvate, polyamines, fatty acid, and starch metabolism helps plants to tolerate cold. *AtSAM1* is responsible for production of S-adenosylmethionine, which promotes the accumulation of polyamines and phytohormone ethylene (Sekula et al., 2020); *AtLIP1* is a triacylglycerol lipases, which negative regulates cold tolerance (Wang et al., 2022); *AtPES1* involves in fatty acid phytyl ester synthesis in chloroplasts (Lippold et al., 2012); *AtCCDC* promotes starch metabolism by interacting with several potential enzyme (Lohmeier-Vogel et al., 2008); *AtBASS2* is a plastidial sodium-dependent pyruvate transporter (Furumoto et al., 2011; Mueller et al., 2014; Lee et al., 2017). Positive selection in above genes leads to non-synonymous substitutions of key amino acid sites that alter enzyme activity, thereby affecting the accumulation of metabolites and promoting environmental adaptation.

These genetic changes were reflected in the differences in the accumulation of components in the two Langdu species. First, *E. fischeriana* reduces the rate of the TCA cycle and maintains low respiration (Close, 1997). At the same time, most amino acid nutrients are reduced to ensure that carbon sources are mostly used to supply protective substances such as glucose, sucrose, raffinose, and xylulose (Figure 6). These soluble sugars are accumulated in large quantities, protecting cell membranes and proteins from freezing and dehydration under cold stress (Klotke et al., 2004). Proline and mannitol also accumulate in *E. fischeriana*, which is due to response to abiotic and biological stresses by affecting osmotic pressure tolerance within plants (Szabados and Savoure, 2010). The accumulation of different metabolites in the roots of two Langdu species may reflect their adaptation to different environments.

Phenotypic formation is the result of both genotype and environmental modification. The phenotype of genuine medicinal materials includes the characteristics, tissue structure, active ingredient composition, and efficacy. The existence of specific genes is the basis for producing specific phenotypes, and the suitable habitat is the driving force for producing specific phenotypes (Yuan and Huang, 2020). Overall, in this study, we detected positive selection genes and metabolites, and these findings will not only shed light on how differentiations between two Langdu species occurs, but also open the door to increased understanding of how plants living in cold environments adapt to different characteristics of high latitude.

### Differences in Active Ingredients of Two Langdu Species Might Lead to Transition in Clinical Usage

Active ingredients, as a part of the phenotype, are also influenced by genotype and environmental modifications. Diterpenoids are regarded as the main bioactive constituents of *E. fischeriana* and *E. ebracteolata*, which can be classified into abietane, tigliane, atisane, pimarane, rosane, kaurane, ingenane, and lathyrane types (Li et al., 2021b; Yang et al., 2021). Among them, abietane type is the main diterpenoid type of the two species. A total of 53 compounds have been isolated from *E. fischeriana*, including jolkinolide B, 17-hydroxyjolkinolide B, jolkinolide A, and 26 compounds from *E. ebracteolate*. In this study, it was found that content of jolkinolide A, jolkinolide B, and jolkinolide E in *E. fischeriana* was 3.1, 36.7, and 2.7 times of that in *E. ebracteolate*, respectively. *DXR* (OG06739) and *MK* (OG07880), involved in diterpenoid biosynthesis, were found in the positive selection library (Ka/Ks > 0.5) mentioned in the previous study, indicating that these two genes had adaptive mutations. Such variation may lead to the changes in enzyme activity and thus becomes more efficient in substrate catalysis, which requires further research. We also found that the most gene expression level of *E. fischeriana* was higher than that of *E. ehracteolata*, which may promote the accumulation of Jolkinolide A/B/E (Figure 7). Moreover, casbane type is unique to *E. ebracteolate*, including Yuexiandajisu A and Yuexiandajisu B (Xu et al., 1998). Casbene is thought to form bicyclic diterpenoids, which in turn forms ingenanes (Luo et al., 2016). A total of 20 ingenanes (ingenol and Esters) have been isolated from *E. ebracteolate* and only 6 from *E. fischeriana*. However, the expression level of *CBS* in the roots of *E. ebracteolate* was significantly higher than that of *E. fischeriana*, and OG17313 was not detected in the latter. We speculated that this was one of the reasons that the content of ingenol in *E. fischeriana* was higher than that in *E. ehracteolata*. However, more definitive evidence will need to be obtained through genome sequencing and biosynthesis pathway analysis. In conclusion, this reflects the different accumulation strategies of the same metabolites in related species, which is the result of long-term adaptation to the environment and also the material basis for the formation of pharmacodynamics.

Jolkinolide B and derivatives in *E. fischeriana* have been proved to have significant anticancer activity (Wang et al., 2009, 2017a). Pharmacological activity of ingenol in *E. ebracteolata* is not clear (Huang et al., 2019), but various esters of ingenol have shown the remarkable biological properties to mimic diacylglycerol and function as endogenous activators of protein kinase (PKC). Furthermore, they were found to have potential in treatment of pancreatic tumor, actinic keratosis (Siller et al., 2009; Parker et al., 2017), and HIV (Johnson et al., 2008). In conclusion, although *E. fischeriana* and *E. ebracteolata* have been used as the same traditional Chinese medicine for a long time, they should have different focuses on anticancer, anti-HIV, and actinic keratosis treatment in clinical use.

### EST-SSRs Are Useful for Population Genetic Analysis Between *Euphorbia*

The development of EST-SSR primers is the further utilization of a large number of EST sequence information and has the characteristics of low cost. *E. fischeriana* and *E. ebracteolata* transcriptome data just provide this data resource. Because EST-SSRs are derived from the relatively conserved transcription part, EST-SSR has a higher translocation between species than genomic SSR, which provides a good tool for studying interspecies population inheritance (Varshney et al., 2005; Kalia et al., 2011). At present, few EST-SSR markers of Langdu have been reported involving *Euphorbia* species (Li et al., 2021a). In this study, 49 of 68 pairs of EST-SSR primers (72.1%) could amplify clear fragments in both Langdu species, 33 pairs (48.5%) were in at least 4 species, 9 were in 8 species, and 2 were in all 9 species (Supplementary Table S6). EST-SSR markers developed in *Euphorbia* showed mobility, indicating that these species may have close genetic relationship and that these markers may have new application value. EST-SSR markers developed based on two Langdu could provide genetic information for research on variety identification, genetic diversity analysis, and molecular marker-assisted breeding.

## Data Availability Statement

The datasets presented in this study can be found in online repositories. The two RNA-seq library datasets (root, stem, and leaf) for this study can be found in the National Center for Biotechnology Information (NCBI) BioProject: PRJNA693977, PRJNA693983.

## Author Contributions

HZ coordinated the project, directed the work, and revised the manuscript. HZ, M-YY, and YH wrote the manuscript. S-FN, F-QL, and HX collected experimental samples. M-YY performed microsection and image processing. M-YY and C-JP performed an in-depth analysis of comparative transcriptome. X-TZ, BT, R-FJ, and KC performed metabolite determination and analysis. L-QH, X-TZ, and YS involved in funding. All authors contributed to the article and approved the submitted version.

## Funding

This research was funded by CACMS Innovation Fund (CI2021A041003), Natural Science Foundation of China (82104342), Fundamental Research Funds of CACMS (ZZ15-YQ-060), and Independent Studies Supported by CACMS (ZK2021001).

## Conflict of Interest

The authors declare that the research was conducted in the absence of any commercial or financial relationships that could be construed as a potential conflict of interest.

## Publisher's Note

All claims expressed in this article are solely those of the authors and do not necessarily represent those of their affiliated organizations, or those of the publisher, the editors and the reviewers. Any product that may be evaluated in this article, or claim that may be made by its manufacturer, is not guaranteed or endorsed by the publisher.
