# Supplementary material for: Comparative Transcriptomics and Metabolites Analysis of Two Closely Related Euphorbia Species Reveal Environmental Adaptation Mechanism and Active Ingredients Difference
Source: Front Plant Sci. 2022 May 31;13:905275. doi: 10.3389/fpls.2022.905275 (PMC9194899; doi:10.3389/fpls.2022.905275)
Supplement: Supplementary file 1 [file Data_Sheet_1.docx]

The method was validated in the aspect of linearity, precision, repeatability, stability and accuracy. A serial appropriate concentration of mixed standard solution were prepared for calibration curves, by plotting the linear regression curve of peak areas (Y) verus standard concentrations (X), and correlation coefficients were calculated. The limits of detection (LODs) and limits of quantification (LOQs) under the present chromatographic condition were determined at S/N (Signal/Noise) of about 3 and 10, respectively. The linear equations, coefficients, linear ranges, LOD and LOQ of quantification of 4 diterpenoids are shown in Table 1.

**Table 1 Linear equations, coefficients, linear ranges, limits of detection and limits of quantification of 4 diterpenoids.**

| Compound | Linear equations | Coefficients | Linear range  (μg/mL) | LOD (ng/mL) | LOQ (ng/mL) |
| --- | --- | --- | --- | --- | --- |
| Jolkinolide A | y=548320x-12063.9 | 0.9998 | 1.36×10^-3^~2.72×10^1^ | 1.36 | 6.8 |
| Jolkinolide B | y=43751.1x-1367.46 | 0.9994 | 2.13×10^-3^~4.26×10^1^ | 2.13 | 10.6 |
| Jolkinolide E | y=26500.5x+231.08 | 0.9996 | 4.2×10^-4^~8.2×10^-1^ | 0.42 | 4.2 |
| Ingenol | y=10497.0x+91.5685 | 0.9995 | 2.04×10^-3^~4.0×10^1^ | 1.96 | 9.8 |

Precision was validated by detecting mixed standard solution six times consecutively, and the relative standard deviations (RSD) were calculated and taken as an evaluation. Results showed an intra-day precision in the range of 2.91-5.21. Six replicates of mixed *E.fischeriana* and *E.ehracteolata* sample solution were prepared and determined under same condition, to test repeatability. The stability test was performed by determining same sample solution at 0 h, 1 h, 2 h, 4 h, 8 h, 12 h and 24 h. The overall repeatability and stability were range from 3.75 to 4.6 and 3.6 to 4.65, respectively.

Accuracy was evaluated in terms of recovery, by adding standard into sample powder whose diterpenoid contents were already known，the three concentrations of high, medium and low were designed, namely 1:1.2, 1:1 and 1:0.8, and each concentration was extracted and quantified in triplicate. Results indicated an overall recovery in the range of 95.9-105.1%, and the RSDs below 5.77.
