# Supplementary material for: Comparative Transcriptomics and Metabolites Analysis of Two Closely Related Euphorbia Species Reveal Environmental Adaptation Mechanism and Active Ingredients Difference
Source: Front Plant Sci. 2022 May 31;13:905275. doi: 10.3389/fpls.2022.905275 (PMC9194899; doi:10.3389/fpls.2022.905275)
Supplement: Supplementary file 2 [file Table_1.DOCX]

**Supplementary Table1 Optimized mass parameters of 4 diterpenoids.**

| **Compound** | **Ion Mode** | **Precursor Ion (m/z)** | **Product Ion (m/z)** | **Q1 Pre**  **(eV)** | **Collision Energy (eV)** | **Q3 Pre**  **(eV)** |
| --- | --- | --- | --- | --- | --- | --- |
| Jolkinolide A | + | 315.00 | 297.10 | -16 | -11 | -14 |
| Jolkinolide B | + | 331.10 | 95.10 | -17 | -21 | -18 |
| Jolkinolide E | + | 301.00 | 173.1 | -16 | -14 | -11 |
| Ingenol | - | 347.25 | 329.20 | 24 | 12 | 12 |
