# Supplementary material for: Comparative Transcriptomics and Metabolites Analysis of Two Closely Related Euphorbia Species Reveal Environmental Adaptation Mechanism and Active Ingredients Difference
Source: Front Plant Sci. 2022 May 31;13:905275. doi: 10.3389/fpls.2022.905275 (PMC9194899; doi:10.3389/fpls.2022.905275)
Supplement: Supplementary file 3 [file Table_2.DOCX]

**Supplementary Table 2 Functional annotation results of *E.fischeriana* and *E.ehracteolata* unigenes.**

|  |  | ***E.fischeriana*** | |  | ***E.ehracteolata*** | |
| --- | --- | --- | --- | --- | --- | --- |
| **Database** |  | Number | Percentage |  | Number | Percentage |
| NR |  | 36204 | 32.18% |  | 38018 | 37.56% |
| NT |  | 22736 | 20.21% |  | 27674 | 27.34% |
| KO |  | 13846 | 12.30% |  | 14235 | 14.06% |
| SwissProt |  | 30073 | 26.73% |  | 27417 | 27.08% |
| PFAM |  | 31585 | 28.07% |  | 26914 | 26.59% |
| GO |  | 32598 | 28.97% |  | 28472 | 28.13% |
| KOG |  | 12696 | 11.28% |  | 9173 | 9.06% |
| All |  | 48033 | 42.70% |  | 44919 | 44.38% |
