# Supplementary material for: Comparative Transcriptomics and Metabolites Analysis of Two Closely Related Euphorbia Species Reveal Environmental Adaptation Mechanism and Active Ingredients Difference
Source: Front Plant Sci. 2022 May 31;13:905275. doi: 10.3389/fpls.2022.905275 (PMC9194899; doi:10.3389/fpls.2022.905275)
Supplement: Supplementary file 4 [file Table_3.DOCX]

**Supplementary Table 3 Partial list of candidate orthologs under positive selection between *E. fischeriana* and *E. ehracteolata*.**

| GO id | GO term | *P*-value | Frequency  in test set | Frequency  in reference set | Orthologous gene | Descriptions | Function |
| --- | --- | --- | --- | --- | --- | --- | --- |
|  | **Biological Process** |  |  |  |  |  |  |
| GO:0015031 | protein transport | 0.02 | 9 | 218 | OG14664, OG06839, OG07794, OG14692, OG17196, OG07291,  OG13657, OG07602, OG03030 | *AtPES1* (Lippold et al., 2012),  *AtGCN1* (Wang et al., 2017),  *TatA* (Walker et al., 1999),  *AtVTI13* (Larson et al., 2014),  *PPR* (Li et al., 2021) | Fatty acid metabolism, cold stress tolerance, root hair development |
| GO:0015886 | heme transport | 0.02 | 3 | 9 | OG13992, OG16428, OG14718 | *At**YLS9* (Griebel et al., 2022) | Cold stress tolerance |
| GO:0051181 | cofactor transport | 0.00 | 3 | 9 | OG13992, OG14718, OG16428 | *AtYLS9* (Griebel et al., 2022) | Cold stress tolerance |
| GO:0071705 | nitrogen compound transport | 0.00 | 4 | 52 | OG16428, OG14718, OG16391, OG13992 | *AtWER1* (Wang et al., 2019),  *AtYLS9* (Griebel et al., 2022) | Root hair development,  cold stress tolerance |
| GO:0071702 | organic substance transport | 0.02 | 34 | 314 | OG14150, OG07253, OG04859, OG07737, OG16515, OG14718,  OG17219, OG07175, OG07288, OG03647, OG03030, OG13754,  OG13657, OG14692, OG07354, OG16702, OG01365, OG07060,  OG13992, OG17193, OG16428, OG14664, OG11159, OG06839,  OG07602, OG09743, OG04971, OG07794, OG07291, OG02209,  OG17196, OG15862, OG04844, OG07033 | *AtPOLD4* (Shultz et al., 2007),  *AtCCDC* (Lohmeier-Vogel et al., 2008),  *AtHSPBP1* (Zhang et al., 2010)*,*  *AtFRL* (Michaels et al., 2004)*,*  *ABC* (Kretzschmar et al., 2011),  *AtLIP1* (Wang et al., 2022),  *AtOST1* (Assmann, 2003),  *AtYLS9* (Griebel et al., 2022),  *AtPES1* (Lippold et al., 2012),  *AtGCN1* (Wang et al., 2017)*,*  *PPR* (Li et al., 2021),  *AtSRP19* (Wang et al., 2008),  *TatA* (Walker et al., 1999)  *AtVTI13* (Larson et al., 2014),  *CPRF2*, *ELIP* (Hayami et al., 2015), *AtBASS2* (Furumoto et al., 2011; Mueller et al., 2014; Lee et al., 2017) | Starch metabolism, heat, cold and drought stresses tolerance,  abiotic and biotic stresses tolerance, glycerolipid metabolism, fatty acid metabolism, root hair development, pyruvate transport |
| GO:0051240 | positive regulation of multicellular organismal process | 0.01 | 2 | 2 | OG06917, OG07011 | *BrRZFP* (Jung et al., 2013)*,*  *TSA1* (Geem et al., 2019) | Cold, salt and dehydration  stresses tolerance |
| GO id | **GO term** | ***P*-value** | **Frequency**  **in test set** | **Frequency**  **in reference set** | **Orthologous gene** | **Descriptions** | **Function** |
| GO:0030522 | intracellular receptor signaling pathway | 0.05 | 2 | 5 | OG07015, OG01198 | *AtPFD* (Perea-Resa et al., 2017)*,*  *VPS54* (Xiang et al., 2013) | Cold stress tolerance,  vesicular trafficking |
| GO:0065004 | protein-DNA complex assembly | 0.03 | 5 | 24 | OG07214, OG15660, OG15240, OG17052, OG16125 | *AtBLT* (Kasili et al., 2011; Mazie and Baum, 2016), *AtSMH* (Hofr et al., 2009),  *BrRZFP* (Jung et al., 2013),  *AtNRP2* (Zhu et al., 2017) | TRICHOME development,  cold stress tolerance,  Root hair development |
| GO:0006461 | protein complex assembly | 0.04 | 12 | 93 | OG16702, OG14718, OG07878, OG13992, OG07214, OG02759,  OG16428, OG16819, OG13999, OG09743, OG13590, OG15659 | *AtBLT*(Kasili et al., 2011; Mazie and Baum, 2016), *AtEML3* (Milutinovic et al., 2019),  *AtMAIL* (de Luxan-Hernandez et al., 2020), *AtPSKR2* (Kaufmann et al., 2021),  *SMH* (Marian et al., 2003) | TRICHOME development,  seed and root development |
| GO:0033014 | tetrapyrrole biosynthetic process | 0.04 | 2 | 30 | OG00941, OG07442 | *AtTIC32* (Hormann et al., 2004) ,  *AtHPPR3* (Xu et al., 2018) | Tyrosine metabolism |
| GO:0019521 | D-gluconate metabolic process | 0.00 | 1 | 11 | OG07442 | *AtHPPR3* (Xu et al., 2018) | Tyrosine metabolism |
| GO:0051188 | cofactor biosynthetic process | 0.04 | 4 | 85 | OG07442, OG13865, OG00941, OG13581 | *AtHPPR3* (Xu et al., 2018),  *AtSAM1* (Sekula et al., 2020),  *AtTIC32* (Hormann et al., 2004),  *AtNAD5* (Qiu et al., 2021) | Tyrosine metabolism,  amino acid biosynthesis,  abiotic and biotic stresses tolerance |
| GO:0006354 | DNA-dependent transcription, elongation | 0.00 | 1 | 3 | OG16324 | *ABC* (Kretzschmar et al., 2011) | abiotic and biotic stresses tolerance |
| GO:0042157 | lipoprotein metabolic process | 0.01 | 5 | 19 | OG14150, OG04859, OG16372, OG07175, OG17193 | *-* | *-* |
| GO:0031123 | RNA 3'-end processing | 0.04 | 3 | 11 | OG04052, OG01293, OG07356 | *AtCSTF64* (Yao et al., 2002)*,*  *AtCPRF1* (Satou et al., 2014)*, AtCFIS1* | Seedling development,  amino acid metabolism |
| GO:0009890 | negative regulation of biosynthetic process | 0.04 | 3 | 11 | OG07740, OG14055, OG15499 | *MUS81* (Enderle et al., 2019),  *RIP* (Zhu et al., 2018),  *NET2A* (Duckney et al., 2021) | DPC repair,  biotic stress tolerance,  pollen development |
| GO:0007050 | cell cycle arrest | 0.03 | 1 | 3 | OG07187 | *AtKRP3*(Jun et al., 2013) | Organ development |
| GO:0044419 | interspecies interaction between organisms | 0.02 | 6 | 134 | OG07349, OG16562, OG07291, OG02170, OG15907, OG06925 | *AtRIE1* (Xu and Li, 2003),  *AtVTI13* (Larson et al., 2014),  *DPB3* (Sato et al., 2015; Sato et al., 2016) | Seed development,  root hair development,  heat stress tolerance |
| GO id | **GO term** | ***P*-value** | **Frequency**  **in test set** | **Frequency**  **in reference set** | **Orthologous gene** | **Descriptions** | **Function** |
|  | **Molecular function** |  |  |  |  |  |  |
| GO:0031490 | chromatin DNA binding | 0.00 | 1 | 1 | OG07565 | *SelH* (Van Hoewyk, 2013) | Selenoproteins and oxidative stresses tolerance |
| GO:0016627 | oxidoreductase activity, acting on the CH-CH group of donors | 0.05 | 3 | 66 | OG00941, OG07822, OG07442 | *AtTIC32* (Hormann et al., 2004),  *HSP,*  *At**HPPR3* (Xu et al., 2018) | Chloroplast development,  tyrosine metabolism |
| GO:0019210 | kinase inhibitor activity | 0.03 | 1 | 6 | OG07187 | *At**KRP3* (Jun et al., 2013) | Organ development |
| GO:0005319 | lipid transporter activity | 0.05 | 1 | 3 | OG07253 | *At**POLD4* (Shultz et al., 2007) | DNA replication |
| GO:0003955 | NAD(P)H dehydrogenase (quinone) activity | 0.01 | 1 | 1 | OG02575 | *At**NQR* (Biniek et al., 2017) | Oxidative stress tolerance |
| GO:0004721 | phosphoprotein phosphatase activity | 0.03 | 1 | 11 | OG01773 | *AtPTP1* (Liu et al., 2016) | Cold stress tolerance |
| GO:0016773 | phosphotransferase activity | 0.03 | 2 | 4 | OG07060, OG07737 | *AtCCDC* (Lohmeier-Vogel et al., 2008) | Starch metabolism |
|  | **Cellular Component** |  |  |  |  |  |  |
| GO:0000428 | DNA-directed RNA polymerase complex | 0.02 | 2 | 16 | OG03552, OG16324 | *AtSCL22* (Ma et al., 2014),  *ABC* (Kretzschmar et al., 2011) | Branch bud formation,  abiotic and biotic stresses tolerance |
| GO:0000347 | THO complex | 0.05 | 1 | 6 | OG07214 | *AtBLT* (Kasili et al., 2011; Mazie and Baum, 2016) | TRICHOME development |
| GO:0031082 | BLOC complex | 0.00 | 1 | 3 | OG07291 | *AtVTI13* (Larson et al., 2014) | Root hair development |
| GO:0030054 | cell junction | 0.00 | 1 | 6 | OG07702 | *EYA* (Musharraf et al., 2008) | *-* |
| GO:0044463 | cell projection part | 0.00 | 2 | 6 | OG16747, OG07793 | *SHY* (Guyon et al., 2004; Wang et al., 2018)*, VPS52* (Xiang et al., 2013) | Pollen development |
| GO:0009357 | transmembrane transporter complex | 0.03 | 2 | 4 | OG07737, OG07060 | *AtCCDC* (Lohmeier-Vogel et al., 2008) | Starch metabolism |

**Reference**

Assmann, S.M. (2003). OPEN STOMATA1 opens the door to ABA signaling in Arabidopsis guard cells. *Trends Plant Sci.* 8(4)**,** 151-153. doi: 10.1016/s1360-1385(03)00052-9.

Biniek, C., Heyno, E., Kruk, J., Sparla, F., Trost, P., and Krieger-Liszkay, A. (2017). Role of the NAD(P)H quinone oxidoreductase NQR and the cytochrome b AIR12 in controlling superoxide generation at the plasma membrane. *Planta* 245(4)**,** 807-817. doi: 10.1007/s00425-016-2643-y.

de Luxan-Hernandez, C., Lohmann, J., Hellmeyer, W., Seanpong, S., Woeltje, K., Magyar, Z., et al. (2020). PP7L is essential for MAIL1-mediated transposable element silencing and primary root growth. *Plant J.* 102(4)**,** 703-717. doi: 10.1111/tpj.14655.

Duckney, P., Kroon, J.T., Dixon, M.R., Hawkins, T.J., Deeks, M.J., and Hussey, P.J. (2021). NETWORKED2-subfamily proteins regulate the cortical actin cytoskeleton of growing pollen tubes and polarised pollen tube growth. *New Phytol.* 231(1)**,** 152-164. doi: 10.1111/nph.17391.

Enderle, J., Dorn, A., Beying, N., Trapp, O., and Puchta, H. (2019). The Protease WSS1A, the Endonuclease MUS81, and the Phosphodiesterase TDP1 Are Involved in Independent Pathways of DNA-protein Crosslink Repair in Plants. *Plant Cell* 31(4)**,** 775-790. doi: 10.1105/tpc.18.00824.

Furumoto, T., Yamaguchi, T., Ohshima-Ichie, Y., Nakamura, M., Tsuchida-Iwata, Y., Shimamura, M., et al. (2011). A plastidial sodium-dependent pyruvate transporter. *Nature* 476(7361)**,** 472-U131. doi: 10.1038/nature10250.

Geem, K.R., Kim, D.H., Lee, D.W., Kwon, Y., Lee, J., Kim, J.H., et al. (2019). Jasmonic acid-inducible TSA1 facilitates ER body formation. *Plant J.* 97(2)**,** 267-280. doi: 10.1111/tpj.14112.

Griebel, T., Schutte, D., Ebert, A., Nguyen, H.H., and Baier, M. (2022). Cold exposure memory reduces pathogen susceptibility in Arabidopsis based on a functional plastid peroxidase system. *Molecular plant-microbe interactions : MPMI*. doi: 10.1094/mpmi-11-21-0283-fi.

Guyon, V., Tang, W.H., Monti, M.M., Raiola, A., De Lorenzo, G., McCormick, S., et al. (2004). Antisense phenotypes reveal a role for SHY, a pollen-specific leucine-rich repeat protein, in pollen tube growth. *Plant J.* 39(4)**,** 643-654. doi: 10.1111/j.1365-313X.2004.02162.x.

Hayami, N., Sakai, Y., Kimura, M., Saito, T., Tokizawa, M., Iuchi, S., et al. (2015). The Responses of Arabidopsis *Early Light-Induced Protein* 2 to Ultraviolet B, High Light, and Cold Stress Are Regulated by a Transcriptional Regulatory Unit Composed of Two Elements. *Plant Physiol.* 169(1)**,** 840-+. doi: 10.1104/pp.15.00398.

Hofr, C., Sultesova, P., Zimmermann, M., Mozgova, I., Schrumpfova, P.P., Wimmerova, M., et al. (2009). Single-Myb-histone proteins from *Arabidopsis thaliana*: a quantitative study of telomere-binding specificity and kinetics. *Biochemical Journal* 419**,** 221-228. doi: 10.1042/bj20082195.

Hormann, F., Kuchler, M., Sveshnikov, D., Oppermann, U., Li, Y., and Soll, J. (2004). Tic32, an essential component in chloroplast biogenesis. *J. Biol. Chem.* 279(33)**,** 34756-34762. doi: 10.1074/jbc.M402817200.

Jun, S.E., Okushima, Y., Nam, J., Umeda, M., and Kim, G.-T. (2013). Kip-Related Protein 3 Is Required for Control of Endoreduplication in the Shoot Apical Meristem and Leaves of *Arabidopsis*. *Mol. Cells* 35(1)**,** 47-53. doi: 10.1007/s10059-013-2270-4.

Jung, Y.J., Lee, I.H., Nou, I.S., Lee, K.D., Rashotte, A.M., and Kang, K.K. (2013). BrRZFP1 a Brassica rapa C3HC4-type RING zinc finger protein involved in cold, salt and dehydration stress. *Plant Biol.* 15(2)**,** 274-283. doi: 10.1111/j.1438-8677.2012.00631.x.

Kasili, R., Huang, C.-C., Walker, J.D., Simmons, L.A., Zhou, J., Faulk, C., et al. (2011). *BRANCHLESS TRICHOMES* links cell shape and cell cycle control in *Arabidopsis* trichomes. *Development* 138(11)**,** 2379-2388. doi: 10.1242/dev.058982.

Kaufmann, C., Stuehrwohldt, N., and Sauter, M. (2021). Tyrosylprotein sulfotransferase-dependent and -independent regulation of root development and signaling by PSK LRR receptor kinases in Arabidopsis. *J. Exp. Bot.* 72(15)**,** 5508-5521. doi: 10.1093/jxb/erab233.

Kretzschmar, T., Burla, B., Lee, Y., Martinoia, E., and Nagy, R. (2011). "Functions of ABC transporters in plants," in *Essays in Biochemistry: Abc Transporters,* ed. F.J. Sharom.), 145-160.

Larson, E.R., Domozych, D.S., and Tierney, M.L. (2014). SNARE VTI13 plays a unique role in endosomal trafficking pathways associated with the vacuole and is essential for cell wall organization and root hair growth in arabidopsis. *Ann. Bot.* 114(6)**,** 1147-1159. doi: 10.1093/aob/mcu041.

Lee, E.-J., Oh, M., Hwang, J.-U., Li-Beisson, Y., Nishida, I., and Lee, Y. (2017). Seed-Specific Overexpression of the Pyruvate Transporter BASS2 Increases Oil Content in Arabidopsis Seeds. *Frontiers in Plant Science* 8. doi: 10.3389/fpls.2017.00194.

Li, X.L., Sun, M.D., Liu, S.J., Teng, Q., Li, S.H., and Jiang, Y.S. (2021). Functions of PPR Proteins in Plant Growth and Development. *Int. J. Mol. Sci.* 22(20). doi: 10.3390/ijms222011274.

Lippold, F., vom Dorp, K., Abraham, M., Holzl, G., Wewer, V., Yilmaz, J.L., et al. (2012). Fatty Acid Phytyl Ester Synthesis in Chloroplasts of *Arabidopsis*. *Plant Cell* 24(5)**,** 2001-2014. doi: 10.1105/tpc.112.095588.

Liu, S., Chen, H., Li, X., and Zhang, W. (2016). A low-temperature-responsive element involved in the regulation of the *Arabidopsis thaliana At1g71850/At1g71860* divergent gene pair. *Plant Cell Rep.* 35(8)**,** 1757-1767. doi: 10.1007/s00299-016-1994-y.

Lohmeier-Vogel, E.M., Kerk, D., Nimick, M., Wrobel, S., Vickerman, L., Muench, D.G., et al. (2008). Arabidopsis At5g39790 encodes a chloroplast-localized, carbohydrate-binding, coiled-coil domain-containing putative scaffold protein. *BMC Plant Biol.* 8. doi: 10.1186/1471-2229-8-120.

Ma, Z., Hu, X., Cai, W., Huang, W., Zhou, X., Luo, Q., et al. (2014). Arabidopsis miR171-Targeted Scarecrow-Like Proteins Bind to GT cis-Elements and Mediate Gibberellin-Regulated Chlorophyll Biosynthesis under Light Conditions. *PLoS Genet.* 10(8). doi: 10.1371/journal.pgen.1004519.

Marian, C.O., Bordoli, S.J., Goltz, M., Santarella, R.A., Jackson, L.P., Danilevskaya, O., et al. (2003). The maize *Single myb histone* 1 gene, Smh1, belongs to a novel gene family and encodes a protein that binds telomere DNA repeats in vitro. *Plant Physiol.* 133(3)**,** 1336-1350. doi: 10.1104/pp.103.026856.

Mazie, A.R., and Baum, D.A. (2016). Clade-specific positive selection on a developmental gene: BRANCHLESS TRICHOME and the evolution of stellate trichomes in Physaria (Brassicaceae). *Molecular Phylogenetics and Evolution* 100**,** 31-40. doi: 10.1016/j.ympev.2016.03.027.

Michaels, S.D., Bezerra, I.C., and Amasino, R.M. (2004). *FRIGIDA*-related genes are required for the winter-annual habit in *Arabidopsis*. *Proc. Natl. Acad. Sci. U. S. A.* 101(9)**,** 3281-3285. doi: 10.1073/pnas.0306778101.

Milutinovic, M., Lindsey, B.E., III, Wijeratne, A., Hernandez, J.M., Grotewold, N., Fernandez, V., et al. (2019). Arabidopsis EMSY-like (EML) histone readers are necessary for post-fertilization seed development, but prevent fertilization-independent seed formation. *Plant Sci.* 285**,** 99-109. doi: 10.1016/j.plantsci.2019.04.007.

Mueller, M., Kunz, H.-H., Schroeder, J.I., Kemp, G., Young, H.S., and Neuhaus, H.E. (2014). Decreased capacity for sodium export out of Arabidopsis chloroplasts impairs salt tolerance, photosynthesis and plant performance. *Plant J.* 78(4)**,** 646-658. doi: 10.1111/tpj.12501.

Musharraf, A., Markschies, N., Teichmann, K., Pankratz, S., Landgraf, K., Englert, C., et al. (2008). Eyes absent proteins: characterization of substrate specificity and phosphatase activity of mutants associated with branchial, otic and renal anomalies. *ChemBioChem* 9(14)**,** 2285-2294. doi: 10.1002/cbic.200800224.

Perea-Resa, C., Rodriguez-Milla, M.A., Iniesto, E., Rubio, V., and Salinas, J. (2017). Prefoldins Negatively Regulate Cold Acclimation in *Arabidopsis thaliana* by Promoting Nuclear Proteasome-Mediated HY5 Degradation. *Molecular Plant* 10(6)**,** 791-804. doi: 10.1016/j.molp.2017.03.012.

Qiu, T., Zhao, X., Feng, H., Qi, L., Yang, J., Peng, Y.-L., et al. (2021). OsNBL3, a mitochondrion-localized pentatricopeptide repeat protein, is involved in splicing *nad5* intron 4 and its disruption causes lesion mimic phenotype with enhanced resistance to biotic and abiotic stresses. *Plant Biotechnol. J.* 19(11)**,** 2277-2290. doi: 10.1111/pbi.13659.

Sato, H., Mizoi, J., Tanaka, H., Maruyama, K., Qin, F., Osakabe, Y., et al. (2015). Arabidopsis DPB3-1, a DREB2A interactor, specifically enhances heat stress-induced gene expression by forming a heat stress-specific transcriptional complex with NF-Y subunits (vol 26, pg 4954, 2014). *Plant Cell* 27(7)**,** 2076-2077. doi: 10.1105/tpc.15.00511.

Sato, H., Todaka, D., Kudo, M., Mizoi, J., Kidokoro, S., Zhao, Y., et al. (2016). The *Arabidopsis* transcriptional regulator DPB3-1 enhances heat stress tolerance without growth retardation in rice. *Plant Biotechnol. J.* 14(8)**,** 1756-1767. doi: 10.1111/pbi.12535.

Satou, M., Enoki, H., Oikawa, A., Ohta, D., Saito, K., Hachiya, T., et al. (2014). Integrated analysis of transcriptome and metabolome of *Arabidopsis albino or pale green* mutants with disrupted nuclear-encoded chloroplast proteins. *Plant Mol. Biol.* 85(4-5)**,** 411-428. doi: 10.1007/s11103-014-0194-9.

Sekula, B., Ruszkowski, M., and Dauter, Z. (2020). S-adenosylmethionine synthases in plants: Structural characterization of type I and II isoenzymes from *Arabidopsis thaliana* and *Medicago truncatula*. *Int. J. Biol. Macromol.* 151**,** 554-565. doi: 10.1016/j.ijbiomac.2020.02.100.

Shultz, R.W., Tatineni, V.M., Hanley-Bowdoin, L., and Thompson, W.F. (2007). Genome-wide analysis of the core DNA replication machinery in the higher plants Arabidopsis and rice(1 W OA ). *Plant Physiol.* 144(4)**,** 1697-1714. doi: 10.1104/pp.107.101105.

Van Hoewyk, D. (2013). A tale of two toxicities: malformed selenoproteins and oxidative stress both contribute to selenium stress in plants. *Ann. Bot.* 112(6)**,** 965-972. doi: 10.1093/aob/mct163.

Walker, M.B., Roy, L.M., Coleman, E., Voelker, R., and Barkan, A. (1999). The maize *tha4* gene functions in Sec-independent protein transport in chloroplasts and is related to *hcf106*, *tatA*, and *tatB*. *J. Cell Biol.* 147(2)**,** 267-275. doi: 10.1083/jcb.147.2.267.

Wang, L., Qian, B., Zhao, L., Liang, M.-H., Zhan, X., and Zhu, J. (2022). Two Triacylglycerol Lipases Are Negative Regulators of Chilling Stress Tolerance in Arabidopsis. *Int. J. Mol. Sci.* 23(6). doi: 10.3390/ijms23063380.

Wang, L.J., Li, H.H., Zhao, C.Z., Li, S.F., Kong, L.Y., Wu, W.W., et al. (2017). The inhibition of protein translation mediated by AtGCN1 is essential for cold tolerance in *Arabidopsis thaliana*. *Plant Cell and Environment* 40(1)**,** 56-68. doi: 10.1111/pce.12826.

Wang, W., Ryu, K.H., Barron, C., and Schiefelbein, J. (2019). Root Epidermal Cell Patterning Is Modulated by a Critical Residue in the WEREWOLF Transcription Factor. *Plant Physiol.* 181(3)**,** 1239-1256. doi: 10.1104/pp.19.00458.

Wang, X., Wang, K., Yin, G., Liu, X., Liu, M., Cao, N., et al. (2018). Pollen-Expressed Leucine-Rich Repeat Extensins Are Essential for Pollen Germination and Growth. *Plant Physiol.* 176(3)**,** 1993-2006. doi: 10.1104/pp.17.01241.

Wang, Y., Zhang, W.-Z., Song, L.-F., Zou, J.-J., Su, Z., and Wu, W.-H. (2008). Transcriptome Analyses Show Changes in Gene Expression to Accompany Pollen Germination and Tube Growth in Arabidopsis. *Plant Physiol.* 148(3)**,** 1201-1211. doi: 10.1104/pp.108.126375.

Xiang, L., Etxeberria, E., and Van den Ende, W. (2013). Vacuolar protein sorting mechanisms in plants. *FEBS J.* 280(4)**,** 979-993. doi: 10.1111/febs.12092.

Xu, J.-J., Fang, X., Li, C.-Y., Zhao, Q., Martin, C., Chen, X.-Y., et al. (2018). Characterization of *Arabidopsis thaliana* Hydroxyphenylpyruvate Reductases in the Tyrosine Conversion Pathway. *Frontiers in Plant Science* 9. doi: 10.3389/fpls.2018.01305.

Xu, R.Q., and Li, Q.S.Q. (2003). A RING-H2 zinc-finger protein gene *RIE1* is essential for seed development in *Arabidopsis*. *Plant Mol. Biol.* 53(1)**,** 37-50. doi: 10.1023/B:PLAN.0000009256.01620.a6.

Yao, Y.L., Song, L.H., Katz, Y., and Galili, G. (2002). Cloning and characterization of *Arabidopsis* homologues of the animal CstF complex that regulates 3 ' mRNA cleavage and polyadenylation. *J. Exp. Bot.* 53(378)**,** 2277-2278. doi: 10.1093/jxb/erf073.

Zhang, J.-X., Wang, C., Yang, C.-Y., Wang, J.-Y., Chen, L., Bao, X.-M., et al. (2010). The role of arabidopsis AtFes1A in cytosolic Hsp70 stability and abiotic stress tolerance. *Plant J.* 62(4)**,** 539-548. doi: 10.1111/j.1365-313X.2010.04173.x.

Zhu, F., Zhou, Y.-K., Ji, Z.-L., and Chen, X.-R. (2018). The Plant Ribosome-Inactivating Proteins Play Important Roles in Defense against Pathogens and Insect Pest Attacks. *Frontiers in Plant Science* 9. doi: 10.3389/fpls.2018.00146.

Zhu, Y., Rong, L., Luo, Q., Wang, B., Zhou, N., Yang, Y., et al. (2017). The Histone Chaperone NRP1 Interacts with WEREWOLF to Activate *GLABRA2* in Arabidopsis Root Hair Development. *Plant Cell* 29(2)**,** 260-276. doi: 10.1105/tpc.16.00719.
