# Supplementary material for: Comparative Transcriptomics and Metabolites Analysis of Two Closely Related Euphorbia Species Reveal Environmental Adaptation Mechanism and Active Ingredients Difference
Source: Front Plant Sci. 2022 May 31;13:905275. doi: 10.3389/fpls.2022.905275 (PMC9194899; doi:10.3389/fpls.2022.905275)
Supplement: Supplementary file 6 [file Table_5.DOCX]

**Supplementary Table 5. Summary of microsatellite loci in *E.fischeriana* and *E.ehracteolata.***

| **Dataset** | **Unigenes containing SSRs** | **SSRs** | **Di-nucleotide repeats** | **Tri-nucleotide repeats** | **Tetra-nucleotide repeats** | **Penta-nucleotide repeats** | **Hexa-nucleotide repeats** | **Mixed repeats** |
| --- | --- | --- | --- | --- | --- | --- | --- | --- |
| ***E.fischeriana*** | 6897 | 8458 | 3724 | 3624 | 406 | 40 | 19 | 645 |
| ***E.ehracteolata*** | 8434 | 10162 | 4522 | 4405 | 417 | 40 | 32 | 746 |
| **ortholog pairs** | 780 | 954 | 293 | 527 | 42 | 1 | 2 | 89 |
| **SSRs (repetitive units >15 bp)** | 113 | 121 | 25 | 66 | 8 | 0 | 0 | 22 |
